# Mongolia health situation: based on the Global Burden of Disease Study 2019

**DOI:** 10.1186/s12889-021-12070-3

**Published:** 2022-01-04

**Authors:** Odgerel Chimed-Ochir, Vanya Delgermaa, Ken Takahashi, Oyuntsetseg Purev, Amarzaya Sarankhuu, Yoshihisa Fujino, Narantuya Bayarmagnai, Otgontuya Dugee, Ryenchindorj Erkhembayar, Battur Lkhagvaa, Chimedsuren Ochir, Tumenjavkhlan Sosorburam, Mohsen Naghavi

**Affiliations:** 1 Department of Public Health and Health Policy, Hiroshima University, Hiroshima, Japan; 2 WHO Representative Office in Mongolia, World Health Organization, Ulaanbaatar, Mongolia; 3 Asbestos Diseases Research Institute, Sydney, NSW Australia; 4 Policy Planning Department, Ministry of Health, Ulaanbaatar, Mongolia; 5 Department of Public Health, Ministry of Health, Ulaanbaatar, Mongolia; 6 Institute of Industrial Ecological Sciences, University of Occupational and Environmental Health, Kitakyushu, Japan; 7 Center for Health Development in Mongolia, Ulaanbaatar, Mongolia; 8 National Public Health Institute, Ministry of Health, Ulaanbaatar, Mongolia; 9 Department of International Cyber Education, Mongolian National University of Medical Sciences, Ulaanbaatar, Mongolia; 10 National Center for Communicable Diseases, Ulaanbaatar, Mongolia; 11 Advisory Board, Ministry of Health, Ulaanbaatar, Mongolia; 12 Public Health Department, For A Healthy Society, Ulaanbaatar, Mongolia; 13 Department of Social Medicine and Health Administration, Tongji Medical College, Ulaanbaatar, Mongolia; 14 Institute for Health Metrics and Evaluation, University of Washington, Seattle, WA USA; 15 Department of Health Metrics Sciences, Subnational Burden of Disease Estimation, Institute for Health Metrics and Evaluation, School of Medicine, University of Washington, 3980 15th Ave. NE, Seattle, WA USA

## Abstract

**Background:**

Over the past few decades, economic, political, and social changes have directly and indirectly affected the health of the Mongolian population. To date, no comprehensive analysis has been conducted on the burden of diseases in this country. Thus, we aimed to describe the leading causes of death and disabling conditions and their trends between 1990 and 2019 in the Mongolian population.

**Methods:**

We used the data from the Global Burden of Disease (GBD) 2019 study. In the current study, we examined life expectancy at birth, healthy life expectancy, the 20 leading causes of death, years of life lost (YLLs), years lived with disability (YLDs), disability-adjusted-life-years (DALYs), and the contribution of major risk factors to DALYs in Mongolia.

**Findings:**

The life expectancy at birth in Mongolia has gradually increased since 1995 and reached 63.8 years for men and 72.7 for women in 2019. The highest increase in the age-standardised death rate between 1990 and 2019 occurred in alcohol use disorders (628.6%; 95% UI 10.0–1109.6) among men, and in liver cancer (129.1%; UI 65.3–222.4) among women. Ischaemic heart disease and stroke showed the highest rates of death, YLLs, and DALYs among both men and women. In 2019, the highest age-standardised rates of DALYs were attributable to high systolic blood pressure and dietary risks.

**Interpretation:**

Although Mongolia saw substantial improvements across many communicable diseases, maternal and neonatal disorders, and under-5 mortality between 1990 and 2019, non-communicable diseases remained leading causes of mortality. The mortality from the most preventable causes such as injury, alcohol use, and dietary risks remain substantially high, suggesting that individual and social efforts are needed to tackle these diseases. Our analyses will support the development of policy priorities and action plans in multiple sectors to improve the overall health of the Mongolian population.

**Funding:**

Bill & Melinda Gates Foundation.

**Supplementary Information:**

The online version contains supplementary material available at 10.1186/s12889-021-12070-3.

## Introduction

Mongolia, located in East Asia with a population of 3.2 million, is the l9^th^ largest and least densely populated country in the world and one-third of the population live in rural areas with a traditional semi-nomadic lifestyle [1]. Mongolia is a lower-middle-income country with an economy based on mining and agriculture. The current health care system in Mongolia was developed in 1921 under the strong, centrally planned Soviet Union Semashko model, with health-care services fully financed by general government revenues. In the early 1990s, with the collapse of the Soviet Union and democratic changes in Mongolia, the Semashko health system was not self-sustaining. Therefore, financing reform with the adoption of the health insurance law in 1993 introduced social health insurance as part of a larger social security system [2]. By 2019, 90.2% of the population was covered by social health insurance [3]. Currently, the health-care service system in Mongolia is characterised by two levels of services, primary and referral-level, and is organised according to the administrative divisions. There are four sources of revenue for the health sector: state budget, health insurance fund, out-of-pocket payments, and international aid and loans [2, 3]. As of 2019, 69.7% of the total health expenditure was financed from the state budget, 26.9% from health insurance funds, and 3.5% from other revenues such as out-of-pocket expenses [3]. 

As observed with other emerging economies, Mongolia has experienced an epidemiological transition resulting in significantly improved health. Average life expectancy at birth has increased, and maternal and neonatal deaths and under-5 mortality have substantially decreased. However, Mongolia still faces a double burden of non-communicable and some communicable diseases [3].

There have been several attempts to evaluate the burden of specific diseases and risks in Mongolia [4, 5]. However, due to the inaccessibility of health data, no comprehensive analysis has been conducted on the burden of disease in this country. Thus, we aimed to describe the leading causes of death and disabling conditions, their trends between 1990 and 2019, and the contribution of major risk factors to disability-adjusted life-years (DALYs) in the Mongolian population. This manuscript was produced as part of the Global Burden of Disease (GBD) Collaborator Network and in accordance with the GBD Protocol.

## Methods

We extracted the data and analyses for Mongolia from the Global Burden of Diseases, Injuries, and Risk Factors (GBD) 2019 study [6]. The GBD study organises causes of death and diseases in a hierarchical list containing four levels in accordance with the International Classification of Diseases (ICD) 9 or 10 codes [7]. In the current study, we examined 20 leading causes of total deaths, years of life lost (YLLs), years lived with disability (YLDs), and DALYs in Mongolia in accordance with the third hierarchical level of classification, which has 169 causes of death. Corresponding ICD-10 codes of causes presented in the current analysis can be found in Additional file 1: Annex 1.

The Cause of Death Ensemble model and Bayesian meta-regression were used to generate estimates of mortality and morbidity by cause for each combination of year, age, and sex; we also show the most recent result (for 2019) for all ages and each sex, along with percentage change from 1990 to 2019. Full results are publicly available online and can be explored with online data visualisation tools and downloaded by using the results query tool [6]. The estimates incorporated data from vital registrations, surveys, and censuses; all data sources used in this analysis are detailed in Additional file 1: Annex 2.

We report all rates as age-standardised rates derived from world population standards that were developed for the GBD study, and each point estimate includes 95% uncertainty intervals (UIs).

YLLs, which represent an estimate of the average years a person would have lived if he or she had not died prematurely, were calculated for each cause by age, sex, and year by multiplying each cause-specific death by the normative standard life expectancy at each age [7]. YLDs, the number of years that an individual lives with a functional impairment caused by a disease, were calculated by multiplying the prevalence of each disease sequela by its disability weight, which was developed using population-based surveys, as described in previous literature [8].

All-cause and cause-specific DALYs, a composite measure of health loss due to both fatal and non-fatal disease burden, were calculated as the sum of YLLs and YLDs for each combination of age, sex, and year in Mongolia.

The relative risk of mortality and morbidity, exposure to each risk factor, and ultimately attributable deaths or DALYs were estimated for each risk-outcome pair. This process is explained in greater detail in the previous literature [9]. Risk factors are organised into five hierarchical levels in the GBD 2019 study [10]. In the current study, we examined the second hierarchical level risk factors in relation to the age-standardised rate of DALYs in Mongolia.

## Results

**Fig. 1:**
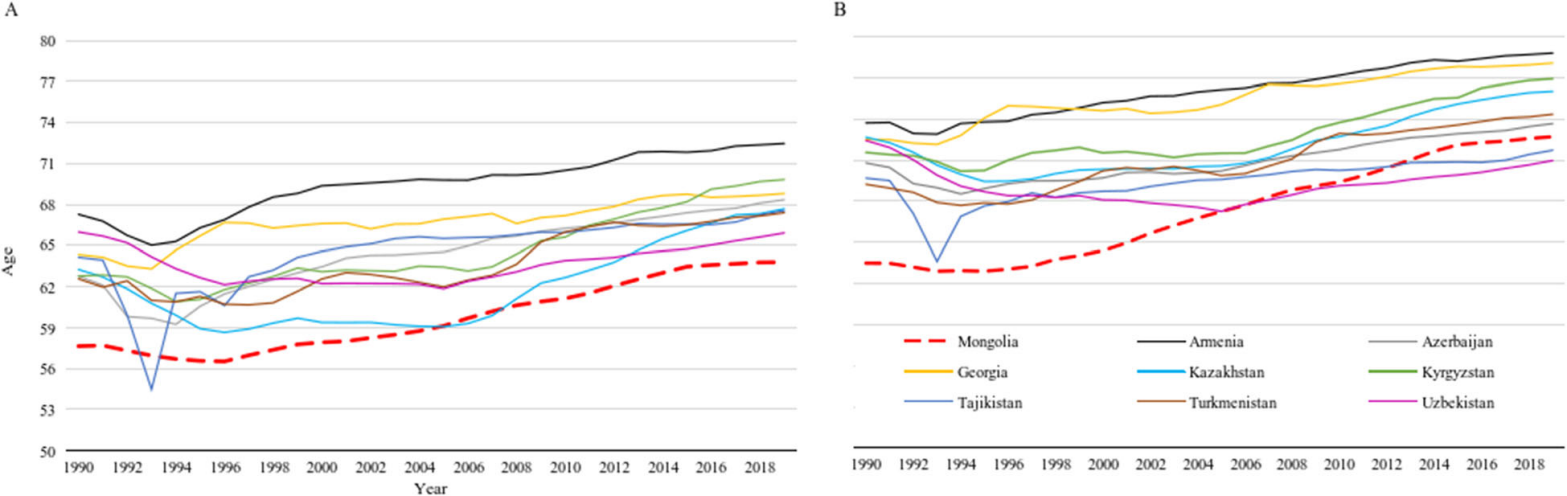
Life expectancy for men (**A**) and women (**B**): Comparison between Mongolia and Central Asian countries

Figure 1 shows that the lowest life expectancy at birth (hereinafter referred to as life expectancy) in Mongolia during 1990–2019 was estimated to be 56.5 (UI 54.6–58.7) years in 1996 for men and 62.9 (UI 60.9–64.9) years in 1995 for women. Since then, life expectancy has gradually increased and reached 63.8 (UI 60.5–66.8) and 72.7 (UI 70.0–75.3) years in 2019 for men and women, respectively. The disparity in male and female life expectancy increased throughout 1990–2019; it was lowest in 1990 (5.9) and highest (8.9) in 2019. Mongolia had the lowest life expectancy for men among Central Asian countries and the third lowest for women after Uzbekistan and Tajikistan. The healthy life expectancy was 57.2 (UI 54.0–60.4) and 63.9 (60.4–67.1) years for men and women in 2019, respectively (Additional file 1: Annex 3).

### Deaths

In Mongolia, under-5 mortality drastically decreased between 1990 (19.7 per 1000 livebirths) and 2019 (3.6 per 1000 livebirths) (Additional file 1: Annex 4).

Table 1 shows the 20 leading causes of death (among Level 3 GBD causes), along with their percentage changes from 1990 to 2019 in Mongolia. Of the total 15,047 deaths of men and 9812 deaths of women due to all causes in 2019, 84.8% (12,757) and 82.8% (8124) were attributed to the 20 leading causes of deaths for men and women, respectively. In particular, ischaemic heart disease, stroke, liver cancer, and cirrhosis and other chronic liver diseases accounted for half of all-cause deaths and had the highest age-standardised mortality rates.

**Table 1:**
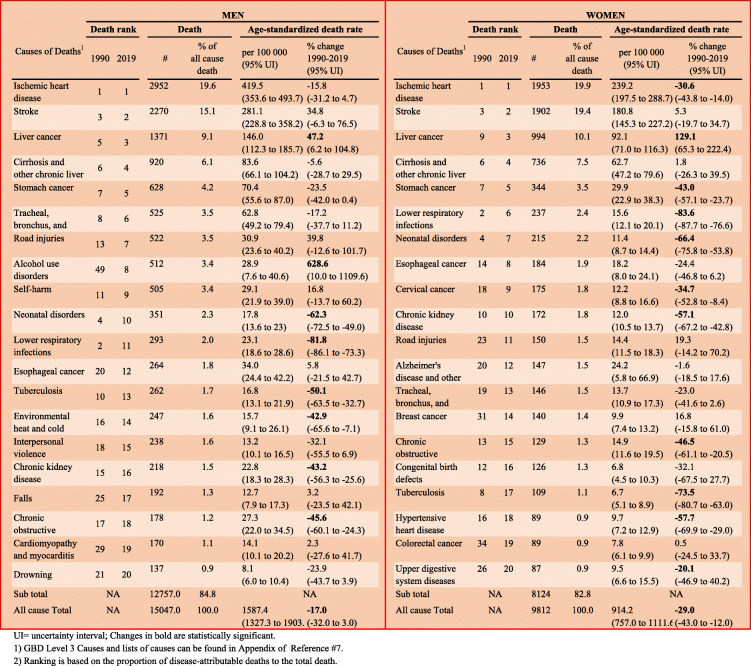
20 leading causes of deaths with percentage changes from 1990 to 2019

UI = uncertainty interval; Changes in bold are statistically significant. 1) GBD Level 3 Causes and lists of causes can be found in Appendix of Reference #7. 2) Ranking is based on the proportion of disease-attributable deaths to the total death

The highest increase in the age-standardised death rate between 1990 and 2019 occurred in alcohol use disorders (628.6%; 95% UI 10.0–1109.6) among men, and in liver cancer (129.1%; 65.3–222.4) among women. By contrast, lower respiratory infections and tuberculosis showed substantial decreases in terms of the age-standardised mortality rate and relative ranking among both men and women during the same period.

### YLLs

Table 2 shows the 20 leading causes of YLLs (Level 3 GBD causes) and their percentage changes from 1990 to 2019 in Mongolia. Of the total 546,994 all-cause YLLs for men in 2019, 83.5% (456964) were attributed to the 20 leading cause of YLLs. For women, of 305,937 all-cause YLLs in 2019, 78.7% (240756) were attributed to the 20 leading causes of YLLs. For both men and women, ischaemic heart disease, stroke, liver cancer, cirrhosis, and neonatal disorders accounted for 45.1% and 49.7% of all-cause YLLs, respectively, and had the highest age-standardised YLL rates in 2019. Infectious diseases, ie, tuberculosis and lower respiratory diseases, showed the greatest decreases for both men and women.

**Table 2:**
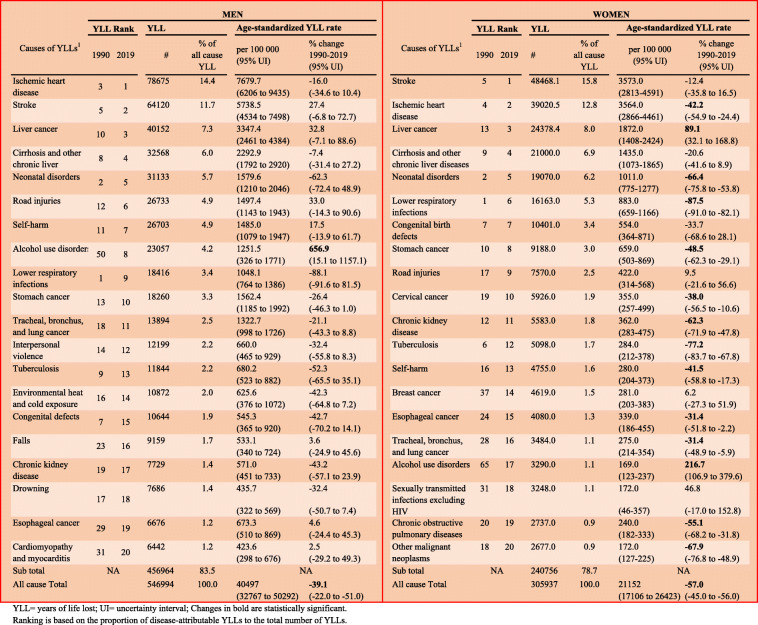
20 leading causes of YLLs with percentage changes from 1990 and 2019

YLL = years of life lost; UI = uncertainty interval; Changes in bold are statistically significant. Ranking is based on the proportion of disease-attributable YLLs to the total number of YLLs

For men, alcohol use disorder showed the largest increase in the age-standardised YLL rate (656.9%; 95% UI 15.1–1157.1) between 1990 and 2019.

For women, among the leading 20 causes, liver cancer showed a significant increase in the age-standardised YLL rate (129.1%; 95% UI 65.3–222.4) between 1990 and 2019.

### DALYs

**Fig. 2:**
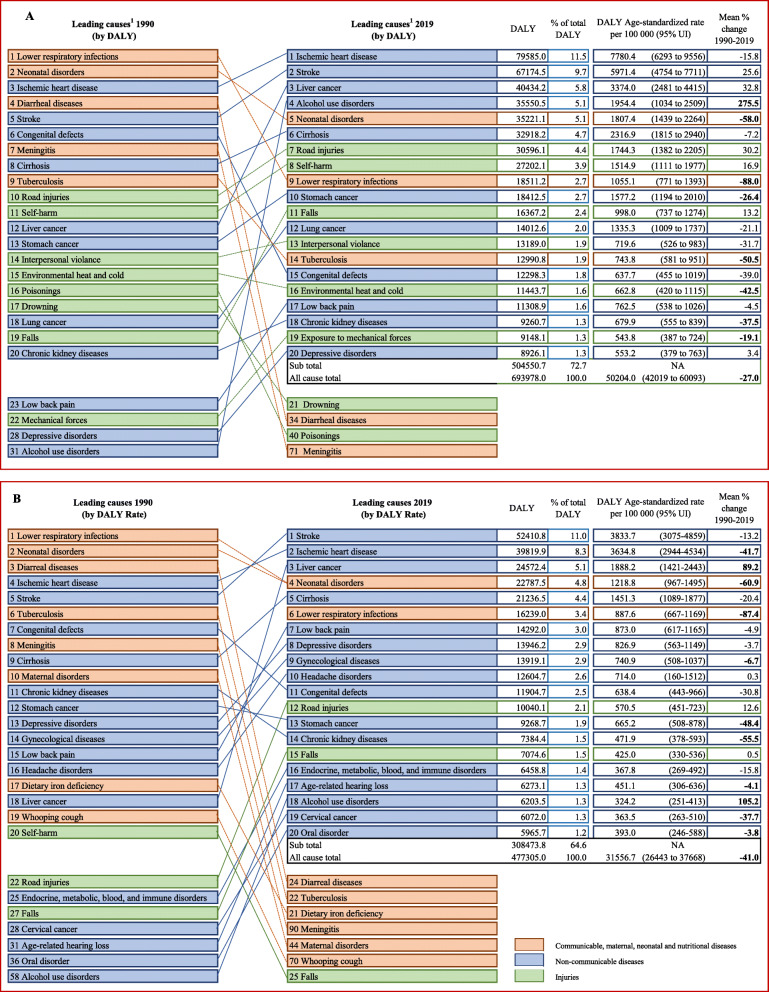
Relative rank of the leading 20 causes of DALYs in 1990 and 2019, number/age-standardized rate in DALY with % changes from 1990 and 2019 for men (**A**) and women (**B**)

Figure 2 shows the 20 leading causes of DALYs and their relative ranks between 1990 and 2019 for men (A) and women (B). Of the total 693,978 all-cause DALYs for men, 72.7% (504,550) were attributed to the 20 leading causes of DALYs. For women, of the 477,305 all-cause DALYs in 2019, 64.6% (308,474) were attributed to the 20 leading causes of DALYs.

Alcohol use disorder showed a significant increase in age-standardised rate for both men (275.5%) and women (105.2%). Liver cancer also increased substantially (89.2%) among women. For both men and women, low back pain and depressive disorders were some of the major causes of disability among the 20 leading causes of DALYs in 2019 (Fig. 2A, B), but these conditions did not substantially contribute to deaths or YLLs.

### Risk

**Fig. 3:**
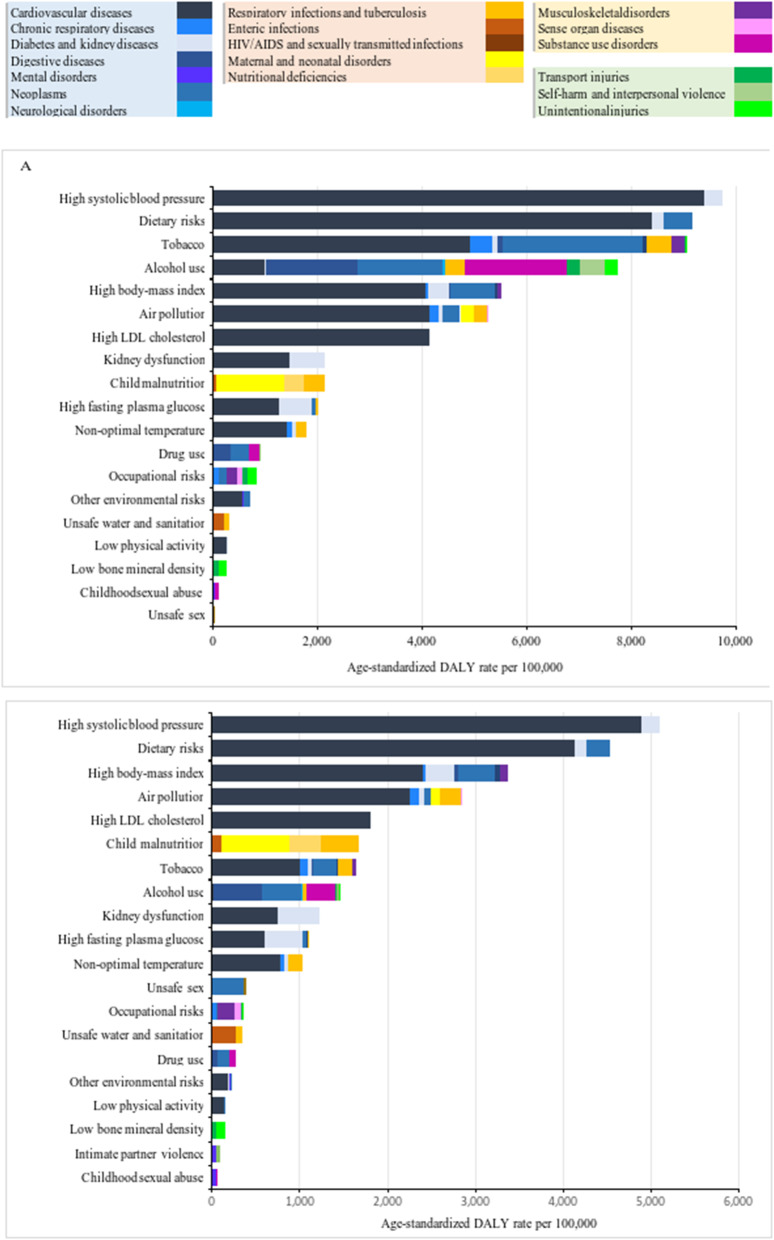
Age-standardized DALY rate attributable to risk factors (GBD Level 2) for men (**A**) and women (**B**) in 2019

Figure 3 shows the age-standardised rates of DALYs for the leading Level 2 risk factors. In 2019, for both men and women, the highest age-standardised rates of DALYs were attributable to high systolic blood pressure (9737 and 5086 DALYs per 100,000, respectively) and dietary risks (9152 and 4524 DALYs per 100,000, respectively). Alcohol use accounted for 7733 DALYs per 100,000) among men and caused the largest number of disease types. Detail numbers and 95%UI can be found in Additional file 1: Annex 5. In further analysis, we showed the burden of the above-mentioned three risk factors (Additional file 1: Annex 6, 7). Alcohol made a huge contribution to alcohol use disorder, liver cancer, and cirrhosis mortality, whereas dietary risks and high systolic blood pressure mainly caused stroke and ischaemic heart disease (Additional file 1: Annex 6). The attributable fraction of total deaths, YLLs, YLDs, and DALYs to alcohol use was much higher (40.5–78.0%) among the 15- to 49-year-old age group. Male-to-female ratios were also much larger (3.4–7.4) for burdens due to alcohol use compared to dietary risks and high systolic blood pressure (Additional file 1: Annex 7).

Table 3 compares the GBD 2019 estimates for Mongolia with Central Asian countries and other groups in terms of the 15 leading causes of death and DALYs in Mongolia for both sexes. The age-standardised rates of death and DALYs for liver cancer were 20 and 17 times higher than global rates, respectively. These differences for cirrhosis were 4 and 3 times, respectively. The rates of DALYs for stomach cancer were 4.0 times higher, and alcohol use disorder was 5.4 times higher than respective global rates.

**Table 3:**
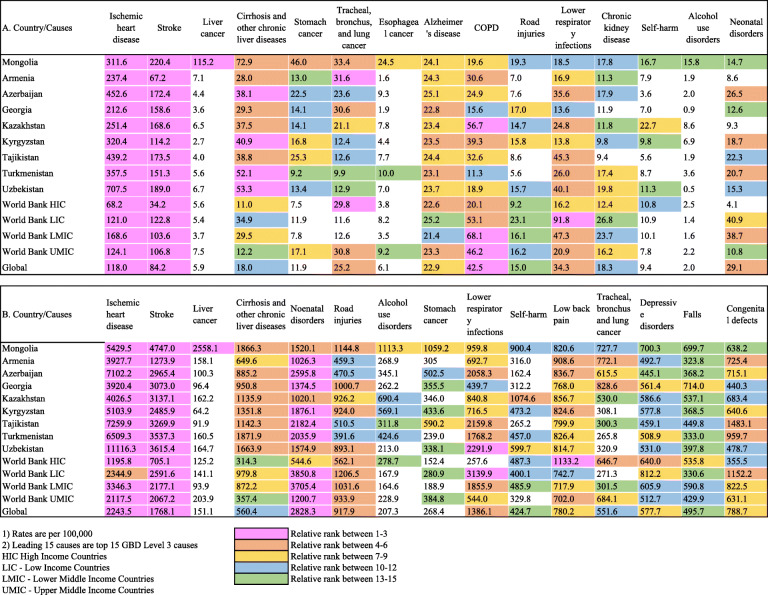
Age-standardized rates of 15 leading causes of deaths (A) and DALYs (B) in Mongolia in 2019 for both sexes, comparison with other countries

1) Rates are per 100,000. 2) Leading 15 causes are top 15 GBD Level 3 causes. HIC High Income Countries. LIC - Low Income Countries. LMIC - Lower Middle Income Countries. UMIC - Upper Middle Income Countries

**Fig. 4:**
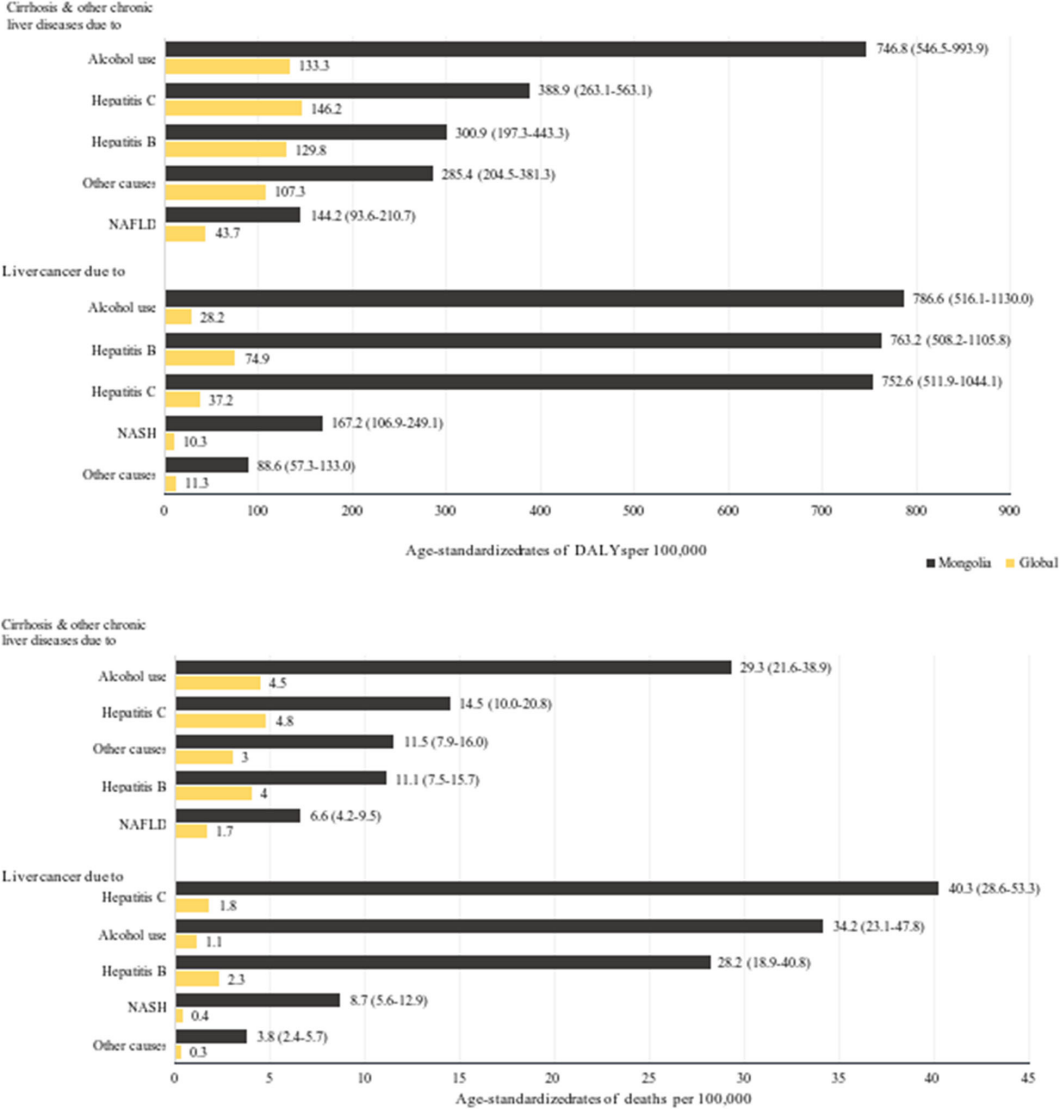
Age-standardized rates of deaths (**A**) and DALYs (**B**) due to liver cancer and cirrhosis (GBD Level 4 Cause) in 2019

Figure 4 compares the death and DALY rates of more specific causes of liver cancer and cirrhosis between Mongolia and the world. Death rates for liver cancer due to alcohol use, hepatitis C, and hepatitis B were 31, 23, and 12 times higher than global rates, respectively.

The death rate for cirrhosis due to alcohol use was 6.5 times higher, and those due to hepatitis C and hepatitis B were three times higher than global rates.

## Discussion

This study is the first comprehensive analysis of disease burden in Mongolia. Over the period 1990 to 2019, Mongolia has experienced an epidemiological transition, as has been observed in many emerging economies. By 2019, rates of some infectious diseases, including lower respiratory infections and tuberculosis, diarrheal diseases, under-5 mortality and neonatal disorders, maternal disorders and some NCDs, such as chronic obstructive pulmonary diseases and chronic kidney diseases, exhibited substantial decreases compared to 1990 among both/either men and/or women. Several NCDs, including ischaemic heart diseases, stroke, cirrhosis, stomach cancer, esophageal cancer and lung cancer, remained leading causes of death from 1990 to 2019, with liver cancer and alcohol use disorder showing a substantial increase in all rates.

During this period, the average life expectancy in Mongolia remarkably improved. The improvement in access to and the quality of healthcare, hygiene, living condition and the successful implementation of various national policies and programs likely contributed to the observed increase of average life expectancy in Mongolia. In particular, national programs in regard of maternal, child and reproductive health that have been successfully implemented since 1990 are believed to have enormous contribution to this achievement.

Since the late 1990s, the successful implementation of various national programs and strategies significantly contributed to the improvement of quality and access to health services, as well as the early detection of diseases, which could have also resulted in a decrease in communicable and some non-communicable disease death and DALY rates. For example, National Strategies in Prevention and Control of HIV/AIDS (Human Immunodeficiency Virus and Acquired Immunodeficiency Syndrome) and tuberculosis have resulted in a decrease of death rate; nonetheless, Mongolia remains one of the nations with the greatest tuberculosis prevalence, with around 4000 tuberculosis cases recorded every year, 10% of which are pediatric [11]. Therefore, tuberculosis prevention, particularly among young people, is critical in Mongolia.

Infectious diseases associated with basic living conditions, such as diarrheal diseases, showed noticeable decrease in its DALY rate. Lower respiratory infections and chronic obstructive pulmonary diseases also had significant decreases in age-standardized death rates between 1990 and 2019, which may have been influenced in part by some policy measures such as the 2013 smoking ban in public places that must have greatly contributed to lessening exposure to passive smoking. However, more research is needed to identify the impact of other risk factors, such as air pollution and indoor air quality, on changes in the burden of these diseases.

For women, the age-standardized mortality and YLL rate of cervical cancer decreased substantially over the study period. Mongolia began early screening of cervical cancer for women aged 24 to 64 in 2011, and coverage reached 46.5% in 2019 [12]. In 2018, the Government of Mongolia also initiated actions to reduce the morbidity and mortality of cervical cancer, such as increased screening coverage, improved diagnosis and treatment capacity, as well as enhanced surveillance. These commitments may have led to the decrease in mortality due to cervical cancer despite its increase in relative ranking and its incidence between 1990 and 2019 [6]. Increases in incidence could be the result of improved early screening or a genuine increase. In any case, earlier prevention is pivotal to improving the cervical cancer situation. A planned nationwide Human Papilloma Virus (HPV) vaccination as of 2024 [13]. will be the promising commitment for cervical cancer prevention. In 2019, the government of Mongolia launched nationwide early screening to prevent from burden of common NCDs [14].

The number of deaths in Mongolia in 2019 was highly skewed, with only five NCDs, including ischaemic heart disease, stroke, liver cancer, cirrhosis, and stomach cancer, accounting for more than half of total deaths for men (54.1%) and women (60.4%). Primary and secondary preventions are of great importance to tackle NCDs. WHO emphasizes four common behavioural risk factors including tobacco use, unhealthy diet, physical inactivity, and harmful use of alcohol and; four metabolic risk factors that increase the risk of NCDs including raised blood pressure, overweight, hyperglycemia and hyperlidipemia [15]. The current study also found that alcohol use, dietary risks, and hypertension were the leading risk factors of death and DALY. According to the national survey on the prevalence of non-communicable diseases and injury risk factors in 2019, one in every four Mongolians and every two males were current smokers in 2019, with 90% of them smoking on a daily basis. One-fifth of the population was physically inactive and 83.4% had daily fruit and vegetable consumption less than the WHO-recommended level of five servings per day [15]. Half of the total population was overweight and one out of every five people was obese. Nearly half of Mongolians (43.4%) had high blood pressure. In general, one-third of the total population had three or more of the aforementioned risk factors [12]. Those living in rural areas had relatively poorer diets with extremely low fiber and high amount of red meat compared to urban population [16]. Mongolians, thus, have considerably high rates of these risks, which pose a high risk of developing NCDs.

Over the last 30 years, stomach cancer, esophageal cancer, and lung cancer remains the leading causes of death. Mongolia has a high prevalence of *Helicobacter pylori* infection, with approximately 70% of patients suffering from gastric problem [17], which significantly increases the risk of stomach cancer [18]. In addition to the risk factors mentioned above, traditional hot tea and meals, a high consumption of red meat [17], re-heated meals prepared the day before and reheated the next day may be considered common risk behavior among Mongolians, which may also contribute to stomach cancer. Among women, the breast cancer death rate did not improve significantly during the study period. This could be due to a lack of knowledge and practice in breast self-examination as well as a low level of healthcare seeking for early detection of breast cancer [12]. Therefore, it heightens the awareness of the improvement for primary prevention programs for all NCDs including cancers.

In Mongolia, most cancers are diagnosed at a late stage, resulting in a low survival rate and a high fatality rate. For example, around 80% of stomach cancer, esophageal cancer and liver cancer, 92% of lung cancer, and 50% of breast cancer were diagnosed at an incurable stage of III or IV [3]. Therefore, secondary prevention measures, such as improving the accessibility and affordability of gastroscopic examination, computer tomography, and mammography, are critical.

The WHO also highlighted the importance of not only primary prevention but also having proper clinical management for the secondary prevention of NCDs as part of its overall portfolio [19]. Mongolia needs to improve the clinical management of NCDs, particularly stroke and ischameic heart disease. In upper-middle- and high-income countries, stroke patients are increasingly managed by stroke units or dedicated centers [20]. Several studies have shown that improved outcomes for acute ischaemic stroke patients are seen when neurocritical care services are available [21]. However, the number of specialized units for acute stroke care in Mongolia is limited, thus, only a small fraction of stroke patients of Mongolia are able to receive timely and adequate specialised emergency care in dedicated centers. In particular, seeking acute stroke care in rural area is very challenging. This condition may contribute to higher mortality rates for stroke. The Government of Mongolia established a second stroke unit in First State Hospital in capital city in 2020 since the first one was established in Third State Hospital in the capital city of Mongolia in 2013 and is working to extend stroke care service nationwide. We anticipate that these efforts, which have been conducted with government resources and the support of international donations, will bring improvements in NCDs in Mongolia in the near future; however, it is important to provide sustainability and formally assess the long-term outcomes of these programmes.

Our study shows that age-standardised death and YLL rates due to alcohol use disorder increased by more than 600% between 1990 and 2019 among men. The highest increase (276%) in DALYs for men occurred in alcohol use disorders. This finding clearly suggests that Mongolians overindulge in alcohol. According to a 2013 national study in Mongolia, roughly 50% of males and 30% of females were current consumers of alcohol, with the greatest alcohol consumption percentage among those aged 25 to 34 years being 52%. In addition, risky drinking practices are well documented in Mongolia; for example, one in every five men has ever driven while under the influence of alcohol, and one-third of current drinkers reported morning drinking [22]. This national survey revealed a high level of knowledge regarding harmful use of alcohol but it also discovered a lack of attitude and practice. It is evident that the consequences of alcohol overuse are major public health and social concerns in Mongolia. For example, previous research found that 72% of violent crime (murder, violent robbery and attacks) is fueled by alcohol in Mongolia, with women and children being especially prone to experiencing daily vodka-fueled domestic violence [23].

In the previous decade, Mongolia’s alcohol sector has grown at an unprecedented rate. The affordability and broad availability of alcohol, a favorable sales environment, strong brand marketing, and its influence on public attitude about alcohol may all contribute to Mongolia’s high prevalence of alcoholism [24]. Therefore, multisectoral cooperation will be essential to tackle alcoholism; efforts could include enforcement of alcoholism prevention laws, policy regulation of the alcohol market (increasing the excise tax on alcohol, improving the implementation and enforcement of the law against alcoholism), promotion of public movements aimed at reducing alcohol consumption, abating underlying causes of heavy alcoholism, i.e., unemployment and poverty, and education of children about excessive alcohol use and health risks.

Mongolia is known to have the world’s highest rate of liver cancer mortality since 1990 [6]. The GBD 2019 study estimated that chronic infections of hepatitis B (HBV) and C (HCV) viruses and alcohol use were the main causes of total death from liver cancers (Fig. 4). This result is consistent with other Mongolian health studies [5, 25, 26]. Alcohol is a confirmed risk factor for liver cancer [27], and its synergetic effect with hepatitis viruses promotes the development of liver disease [28]. Thus, Mongolians became more vulnerable to liver cancer and cirrhosis with the earlier endemic of chronic infections of HBV and HCV as well as heavy alcoholism.

Until the 1990s, reusable glass syringes and needles were used in the medical practices of Mongolia, home injection with reusable syringes and unsafe blood transfusion was quite common [29]. These circumstances could have caused the high prevalence of HBV and HCV.

Since then, the use of disposable syringes has expanded in the medical practice of Mongolia. Moreover, a national HBV vaccination program for children was initiated in 1992, with the most recent coverage rate within 24 hours after birth reaching 98% [30]. We believed that the effects of these changes on cohorts born after 1992 would likely reduce the overall prevalence of HCV- and HBV-induced liver cancer. However, during 1990 and 2019, the incidence of acute hepatitis B infection decreased by 40%, whereas no improvement was evident in acute hepatitis C infection [6]. The diagnostic capability of hepatitis virus detection has advanced considerably in Mongolia since 2005. In addition, assurance of the sterilisation of equipment used in small private clinics, especially dental clinics and those handling cosmetic procedures, is still challenging [31]. The aforementioned reasons may have contributed to the lack of improvement in incidence of acute hepatitis C infection.

Mongolia initiated a Healthy Liver National Program during 2017–2020, which sought to eliminate cancer-causing HCV and HBV and reduce mortality related to liver cirrhosis and liver cancer [32]. This programme has the largest budget from government funding committed for any single health programme in Mongolia, at approximately MNT 166 billion (US$40 million), over 4 years of programme implementation [33]. Within the scope of this programme, the government supported laboratory tests for detection of viruses and viral load, and treatment for HBV and HCV. As of May 2020, 52% of the target population had been tested for HCV and HBV, and 50% of the total budget had been used [33]. The program warrants a comprehensive assessment upon its completion in regards to its goals. Continuation of this programme has been reflected in the government’s action plan for 2020–2024 [34]. These initiatives therefore offer hope for improving the status of liver disease in the coming decades.

As earlier mentioned, although Mongolia showed great improvement in average life expectancy, the life expectancy of women has long been much higher than that of men; this gap reached 9 years in 2019, giving Mongolia one of the highest sex gaps in life expectancy worldwide [6].

Indeed, various explanations could account for the sex-based difference in life expectancy in Mongolia, including occupational, metabolic, and environmental risk factors. For example, age-standardised rates of death and YLLs due to alcohol and drug use are 210% and 444% higher in men than in women, respectively. These rates due to occupational risks are much higher (447% to 584%) in men than in women (Additional file 1: Annex 8). Mongolian researchers also previously reported that men had higher risks of occupational diseases including occupational respiratory diseases, musculoskeletal disorder, cardiovascular diseases, and hearing loss in coal mining and construction sector [35].

In addition, health-seeking behaviour, health literacy, and education level may have contributed to the difference in life expectancy. Men tend to seek health care much less than women in Mongolia. Higher education levels can also reduce mortality [36], making it notable that Mongolian families typically prioritise education for daughters over sons. As of 2017, more than 60% of university graduates in Mongolia were women [37]. Predominance of female workers in certain jobs is evident in Mongolia [37].

In 2019, women were more likely to have more DALYs due to many disabling conditions that predominantly lead to YLDs (Additional file 1: Annex 9) but do not cause substantial death, such as low back pain, headache disorder, and depressive disorders. By contrast, injuries (e.g., road accidents, self-harm, falls, and interpersonal violence) exhibited high rates of death and YLLs among men and greatly contributed to DALYs in men. The National Trauma Study of Mongolia also revealed in 2018 that men are more prone to injuries compared to women [38]. The incidence of road injuries are greater in rural areas where the infrastructure is poor whereas fall injuries are higher in the capital city [38]. Therefore, these characteristics of injuries need to be well considered for the development of policy.

Although it is expensive to manage diseases such as neurological disorders and cancers, it is relatively feasible to manage alcohol use disorder and injury-related deaths. Thus, it seems possible that the burden of diseases in Mongolia could be largely improved through managing these risks.

The limitations of study include those described for the whole GBD 2019 study [7–10]. The most challenging issue worth mentioning is insufficient comprehensive vital registration data for Mongolia, thus, most of the results presented for Mongolia came from regional patterns and covariates, with few data points available to guide estimation. Non-fatal disease estimations (YLD) fully relied on estimation technique due to lack of hospital data from Mongolia. Thus, it may have caused the wide range of uncertainty intervals for some estimations e.g., alcohol use disorder that may affect the validity of result. Therefore, health policy makers and relevant authorities of Mongolia should have concern of both unavailability of data and quality of reported data, and be aware of the significance of incorporating a country specific data sources into the GBD databank to improve the accuracy of estimation. Although we herein discuss the burden of diseases in Mongolia at the national level per GBD 2019 estimates, the small population of Mongolia within the large territory, huge disparities between rural and urban area in people’s lifestyle, infrastructure, availability of and access to health service mean that the country’s health patterns have a substantial diversity that would be better captured at the subnational level.

## Conclusion

Although Mongolia saw substantial improvements across many communicable diseases, some NCDs, and maternal and neonatal diseases between 1990 and 2019, NCDs including ischaemic heart diseases, stroke, cirrhosis, and stomach cancer remained leading causes of death, with liver cancer and alcohol use disorder showing a substantial increase in all rates. It warrants future research to thoroughly investigate the potential solutions of reducing the leading NCDs, evaluate past and current NCD-related programmes in Mongolia, and seek the facilitation of developing evidence-based health policies. In addition, mortality from the most preventable causes and risks, such as injury, alcohol use, and dietary risks, remain substantially high, suggesting that individual and/or social efforts are needed to tackle these diseases. The small population of Mongolia within the large territory, huge disparity between rural and urban lifestyle, infrastructure, availability of and access to health service mean that the country’s health patterns have a substantial diversity that would be better captured at the subnational level in the next step. The current government policies of Mongolia for NCDs and essential healthcare services align with the GBD 2019 results for Mongolia, however, our analyses will further support the development of policy priorities and action plans in multiple sectors to improve the health of the Mongolian population.

## Supplementary Information

**Additional file 1.**

## Data Availability

The datasets supporting the conclusions of this article are available from the corresponding author upon request.
