# Supplementary material for: Mongolia health situation: based on the Global Burden of Disease Study 2019
Source: BMC Public Health. 2022 Jan 4;22:5. doi: 10.1186/s12889-021-12070-3 (PMC8729000; doi:10.1186/s12889-021-12070-3)
Supplement: Supplementary file 1 — Additional file 1. [file 12889_2021_12070_MOESM1_ESM.pdf]

Annex 1. List of International Classification of Diseases (ICD) codes mapped

Disease	ICD Code
Age-related and other hearing loss	H71-H75.83, H80-H80.93, H83-H83.93, H90-H91, H91.1-H91.93, H94-H94.83, Q16-Q16.9
Alcohol use disorders	E24.4, F10-F10.99, G31.2, G62.1, R78.0, X45-X45.9, X65-X65.9, Y15-Y15.9, Z81.1
Alzheimer's disease and other dementias	F00-F02.0, F02.8-F03.91, F06.2, G30-G31.1, G31.8-G32.89
Anxiety disorders	F40-F44.9, F93-F93.2
Bipolar disorder	F30-F31.9, F34.0
Blindness and vision impairment	H25-H28.8, H31-H36.8, H40-H40.9, H42-H42.8, H46-H54.9
Breast cancer	C50-C50.629, C50.8-C50.929, Z12.3-Z12.39, Z80.3, Z85.3, Z86.000
Cardiomyopathy and myocarditis	B33.2-B33.20, B33.22-B33.24, D86.85, I40-I41.8, I42-I43.8, I51.4-I51.6
Cervical cancer	C53-C53.9, Z12.4, Z85.41
Chronic kidney disease	D63.1, E08.2-E08.29, E10.2-E10.29, E11.2-E11.29, E12.2, E13.2-E13.29, E14.2, I12-I13.9, N02-N08.8, N15.0, N17-N19, Q60-Q63.2, Q63.8-Q63.9, Q64.2-Q64.9, Z49-Z49.32, Z52.4, Z99.2
Chronic obstructive pulmonary disease	J41-J42.4, J43-J44.9
Cirrhosis and other chronic liver diseases	I85-I85.9, I98.2, K70-K71, K71.3-K72, K72.1-K75, K75.2, K75.4-K76.2, K76.4-K77.8, R16-R18.9, Z52.6, Z94.4
Colon and rectum cancer	C18-C19.0, C20, C21-C21.8, Z12.1-Z12.13, Z85.03-Z85.048, Z86.010
Congenital birth defects	G71.2, P96.0, Q00-Q07.9, Q10-Q15.9, Q17-Q18.9, Q20-Q28.9, Q30-Q45.9, Q50-Q56.4, Q63.3, Q64-Q64.19, Q65-Q87.89, Q89-Q89.8, Q90-Q93.9, Q95-Q99.9, Z13.7-Z13.79, Z14-Z15.89, Z82.7-Z82.79, Z87.7-Z87.798
Depressive disorder	F32-F33.9, F34.1
Dermatitis	L20-L23.2, L23.4-L27, L27.2-L27.9, L30-L30.2, L30.5-L30.9
Diabetes mellitus	E08-E08.11, E08.3-E08.9, E10-E10.11, E10.3-E11.1, E11.3-E12.1, E12.3-E13.11, E13.3-E14.1, E14.3-E14.9, R73-R73.9, Z13.1, Z83.3
Diarrheal diseases	A00-A00.9, A02-A02.0, A02.8-A07, A07.2-A07.4, A08-A08.8, A09, K52.1, Z22.1, Z23.0
Dietary iron deficiency	D50-D50.9
Drowning	W65-W70.9, W73-W74.9
Drug use disorders	F11-F19.99, P96.1, R78.1-R78.9, Z81.2-Z81.4
Endocrine, metabolic, blood, and immune disorders	C7A00-C7B8, D3A00-D3A8, D66-D69.49, D69.6-D70.0, D70.4-D77, D80-D84.9, D86.8, D86.82-D86.84, D86.86-D86.89, D89-D89.2, D89.8, D89.82-D89.9, E03-E03.1, E03.3-E06.3, E06.5-E07.9, E15-E16, E16.1-E16.9, E20-E23.0, E23.2-E24.1, E24.3, E24.8-E27.2, E27.4-E28.1, E28.3-E32.9, E34, E34.1-E35.8, E65-E66.09, E66.2-E68, E70-E80.09, E80.3-E85.9, E88-E88.9, E90-E998, Z83.4-Z83.49
Environmental heat and cold exposure	L55-L55.9, L58-L58.9, W88-W94.9, W97.9, W99-W99.9, X30-X32.9, X39-X39.9
Esophageal cancer	C15-C15.9, Z85.01
Exposure to mechanical forces	W20-W38.9, W40-W43.9, W45.0-W45.2, W46-W46.2, W49-W52
Falls	W00-W19.9
Fire, heat, and hot substances	X00-X06.9, X08-X19.9
Foreign body	H02.81-H02.819, H44.6-H44.799, M60.2-M60.28, W44-W45, W45.3-W45.9, W75-W76.9, W78-W80.9, W83-W84.9
Gynecological diseases	B37.3-B37.49, D25-D26, E28.2, N61-N64.9, N72-N72.0, N75-N77.8, N80-N81.9, N83-N84, N84.2-N86, N88-N95.9, N97-N98.9, R30-R37, R39-R39.9, R87-R87.9, Z01.4-Z01.7, Z31-Z31.9, Z86.1-Z86.19, Z87.4-Z87.448
Headache disorder	G43-G44.89
Hypertensive heart diseases	I11-I11.2, I11.9
Idiopathic epilepsy	G40-G41.9, Z82.0
Interpersonal violence	T74.2-T76.22, X85-Y08.9, Y87.1-Y87.2
Ischemic heart disease	I20-I21.6, I21.9-I25.9, Z82.4-Z82.49
Liver cancer	C22-C22.4, C22.7-C22.9, Z85.05
Low back pain	G54.4, M47.015-M47.019, M47.15-M47.18, M47.25-M47.28, M47.815-M47.818, M47.896-M47.899, M48.05-M48.08, M48.16-M48.19, M48.25-M48.27, M48.35-M48.38, M48.45-M48.48, M48.55-M48.58, M49.85-M49.88, M51.05-M51.07, M51.15-M51.17, M51.25-M51.27, M51.35-M51.37, M51.45-M51.47, M51.85-M51.87, M53.3, M53.85-M53.88, M54.05-M54.09, M54.15-M54.18, M54.3-M54.5, M99.03-M99.04, M99.13-M99.14, M99.23-M99.24, M99.33-M99.34, M99.43-M99.44, M99.53-M99.54, M99.63-M99.64, M99.73-M99.74, M99.83-M99.84
Lower respiratory infections	A48.1, A70, B96.0-B96.1, B97.21, B97.4-B97.6, J09-J18.2, J18.8-J18.9, J19.6-J22.9, J85.1, J91.0, P23-P23.9, U04 -U04.9, Z25.1
Neck pain	G54.2, M47.011-M47.013, M47.11-M47.13, M47.21-M47.23, M47.811-M47.813, M47.892-M47.894, M48.01-M48.03, M48.12-M48.14, M48.21-M48.23, M48.31-M48.33, M48.41-M48.43, M48.51-M48.53, M49.81-M49.83, M50-M50.93, M53.0-M53.1, M53.81-M53.83, M54.01-M54.03, M54.11-M54.13, M54.2, M54.81, M99.01, M99.11, M99.21, M99.31, M99.41, M99.51, M99.61, M99.71, M99.81

Neonatal disorders	A40.1, B95.1, P00-P05.9, P07-P15.9, P19-P22.9, P24-P29.9, P36-P36.9, P38-P39.9, P50-P61.9, P70-P72.9, P74-P74.9, P75.0-P78.9, P80-P81.9, P83-P84, P90-P92.9, P94-P94.9, P96, P96.3-P96.4, P96.8-P96.9, P99.9
Oral disorders	K00-K08.499, K08.8-K14.9, M26-M27.9, Z01.2-Z01.21, Z13.84, Z96.5
Osteoarthritis	M16-M18.9
Other malignant cancers	C17-C17.9, C30-C31.9, C37-C38.8, C40-C41.9, C47-C4A, C51-C52.9, C57-C57.8, C58-C58.0, C60-C60.9, C63-C63.8, C66-C66.9, C68.0-C68.8, C69-C69.9, C74-C75.8, D07.4, D09.2, D13.2-D13.3, D14.0, D15-D16.9, D28.0-D28.1, D28.7, D29.0, D30.2, D30.4-D30.8, D31-D31.9, D35-D35.2, D35.5-D36, D36.1-D36.7, D37.2, D38.2-D38.5, D39.2, D39.8, D41.2-D41.3, D44.1-D44.8, D48.0-D48.4
Other mental disorders	F04-F06.1, F06.3-F07.0, F08-F09.9, F21-F24, F26-F29.9, F34, F34.8-F34.9, F38-F39, F45-F49, F51-F52.9, F55-F55.8, F56-F69.0, F80-F89.0, F93.3-F99.0, G47-G47.29, G47.4-G47.9, R40-R40.4, R45-R46.89, R55-R55.0, Z03.2, Z04.6-Z04.72, Z13.4, Z64, Z81, Z81.8, Z86.5-Z86.59
Other muscoloskeletal disease	G54.3, I27.1, L93-L93.2, M00-M03.6, M06-M07.69, M11-M15.9, M19-M19.93, M20-M25.9, M30-M32.9, M34-M36.8, M40-M43.9, M45-M47.01, M47.014, M47.02-M47.10, M47.14, M47.2-M47.20, M47.24, M47.8-M47.81, M47.814, M47.819-M47.891, M47.895, M47.9-M48.00, M48.04, M48.1-M48.11, M48.15, M48.2-M48.20, M48.24, M48.3-M48.30, M48.34, M48.4-M48.40, M48.44, M48.5-M48.50, M48.54, M48.8-M49, M49.2-M49.80, M49.84, M49.89, M51-M51.04, M51.1-M51.14, M51.2-M51.24, M51.3-M51.34, M51.4-M51.44, M51.8-M51.84, M51.9, M53, M53.2, M53.8-M53.80, M53.84, M53.9-M54.00, M54.04, M54.1-M54.10, M54.14, M54.6-M54.8, M54.89-M54.9, M61-M63.89, M65-M68.8, M70-M72.4, M72.8-M73, M75-M77.9, M79-M79.676, M79.8-M87.09, M87.2-M89.59, M89.7-M95.9, M99-M99.00, M99.02, M99.05-M99.10, M99.12, M99.15-M99.20, M99.22, M99.25-M99.30, M99.32, M99.35-M99.40, M99.42, M99.45-M99.50, M99.52, M99.55-M99.60, M99.62, M99.65-M99.70, M99.72, M99.75-M99.80, M99.82, M99.85-M99.9
Road injuries	V01-V04.99, V06-V80.929, V82-V82.9, V87.2-V87.3
Schizophrenia	F20-F20.9, F25-F25.9
Self-harm	X60-X64.9, X66-X84.9, Y87.0
Sexually transmitted infections excluding HIV	A50-A60.9, A63-A64.0, B63, I98.0, K67.0-K67.2, M73.0-M73.8, N70-N71.9, N73-N74, N74.2-N74.8, Z11.3, Z20.2, Z22.4
Stomach cancer	C16-C16.9, Z12.0, Z85.02-Z85.028
Stroke	G45-G46.8, I60-I62, I62.9-I64, I64.1, I65-I69.998, Z82.3
Tracheal, bronchus, and lung cancer	C33, C34-C34.92, Z12.2, Z80.1-Z80.2, Z85.1-Z85.20
Tuberculosis	A10-A14, A15 -A18.89, A19-A19.9, B90-B90.9, K67.3, K93.0, M49.0, N74.0-N74.1, P37.0, U84.3, Z03.0, Z11.1, Z20.1, Z23.2
Upper digestive system diseases	K21-K21.9, K22.7-K22.719, K25-K30, R12
Vitamin A deficiency	E50-E50.9, E64.1

Annex 2. Data source used for estimation of burden of diseases in Mongolia

1. Allen RW, Gombojav E, Barkhasragchaa B, Byambaa T, Lkhasuren O, Amram O, Takaro TK, Janes CR. An assessment of air pollution and its attributable mortality in Ulaanbaatar, Mongolia. Air Qual Atmos Health. 2013; 6(1): 137-50 as it appears in Shaddick G, Thomas ML. Particulate Matter 2.5 and 10 Surface Monitor Station Expanded Database 2008-2017. [Unpublished]
2. Baasanhu J, Johnson GJ, Burendei G, Minassian DC. Prevalence and causes of blindness and visual impairment in Mongolia: a survey of populations aged 40 years and older. Bull World Health Organ. 1994; 72(5): 771-6
3. Baatarkhuu O, Kim DY, Ahn SH, Nymadawa P, Dahgwahdorj Y, Shagdarsuren M, Park JY, Choi JW, Oyunbileg J, Oyunsuren T, Han KH. Prevalence and genotype distribution of hepatitis C virus among apparently healthy individuals in Mongolia: a population-based nationwide study. Liver Int. 2008; 28(10): 138995
4. Bayasgalan G, Naranbat D, Tsedmaa B, Tsogmaa B, Sukhee D, Amarjargal O, Lhagvasuren T, Radnaabazar J, Rowe PJ. Clinical patterns and major causes of infertility in Mongolia. J Obstet Gynaecol Res. 2004; 30(5): 386-93
5. Bellizzi S, Ali MM, Abalos E, Betran AP, Kapila J, Pileggi-Castro C, Vogel JP, Merialdi M. Are hypertensive disorders in pregnancy associated with congenital malformations in offspring? Evidence from the WHO Multicountry cross sectional survey on maternal and newborn health. BMC Pregnancy Childbirth. 2016; 16(1): 198
6. Bhutani VK, Zipursky A, Blencowe H, Khanna R, Sgro M, Ebbesen F, Bell J, Mori R, Slusher TM, Fahmy N, Paul VK, Du L, Okolo AA, de Almeida MF, Olusanya BO, Kumar P, Cousens S, Lawn JE. Neonatal hyperbilirubinemia and Rhesus disease of the newborn: incidence and impairment estimates for 2010 at regional and global levels. Pediatr Res. 2013; 74(Suppl 1): 86-100
7. BP. BP Statistical Review of World Energy 2016. London, United Kingdom: BP, 2016
8. British Geological Survey, Natural Environment Research Council. World Mineral Production 2007-2011. Nottingham, United Kingdom: British Geological Survey, Natural Environment Research Council, 2013
9. Centers for Disease Control and Prevention (CDC) and World Health Organization (WHO). Mongolia Global Youth Tobacco Survey 2003. Atlanta, United States: Centers for Disease Control and Prevention (CDC)
10. Centers for Disease Control and Prevention (CDC) and World Health Organization (WHO). Mongolia Global Youth Tobacco Survey 2007. United States: Centers for Disease Control and Prevention (CDC), 2007
11. Centers for Disease Control and Prevention (CDC), Ministry of Health (Mongolia), United Nations Children's Fund (UNICEF), World Health Organization (WHO). Mongolia Survey Assessing the Nutritional Consequences of the Dzud 2001
12. Centers for Disease Control and Prevention (CDC), National Center for Public Health (Mongolia), World Health Organization (WHO). Mongolia Global Youth Tobacco Survey 2014. Atlanta, United States of America: Centers for Disease Control and Prevention (CDC)
13. Centers for Disease Control and Prevention (CDC), Public Health Institute, Ministry of Health (Mongolia), World Health Organization (WHO). Mongolia Global School-Based Student Health Survey 2010
14. Centers for Disease Control and Prevention (CDC), World Health Organization (WHO). Mongolia Global School-Based Student Health Survey 2013
15. Chitsulo L, Engels D, Montresor A, Savioli L. The global status of schistosomiasis and its control. Acta Trop. 2000; 77(1): 41-51
16. Cystic Echinococcosis Endemicity Estimates identified through systematic review and personal communication, as provided by GBD 2015 expert. [Unpublished]
17. Dananché C, Sánchez Picot V, Bénet T, Messaoudi M, Chou M, Wang J, Pape J-W, Awasthi S, Bavdekar A, Rakoto-Andrianarivelo M, Sylla M, Nymadawa P, Russomando G, Komurian-Pradel F, Endtz H, Paranhos-Baccalà G, Vanhems P, For The Gabriel Network null. Burden of Influenza in Less Than 5-Year-Old Children Admitted to Hospital with Pneumonia in Developing and Emerging Countries: A Descriptive, Multicenter Study. Am J Trop Med Hyg. 2018; 98(6): 1805–10
18. Davaalkham D, Ojima T, Nymadawa P, Tsend N, Lkhagvasuren T, Wiersma S, Uehara R, Watanabe M, Oki I, Nakamura Y. Seroepidemiology of hepatitis B virus infection among children in Mongolia: results of a nationwide survey. Pediatr Int. 2007; 49(3): 368-74
19. Development Economics Data Group (DECDG), World Bank, National Statistical Office of Mongolia. Mongolia Population and Housing Census 1963
20. Development Economics Data Group (DECDG), World Bank, National Statistical Office of Mongolia. Mongolia Population and Housing Census 1969
21. Development Economics Data Group (DECDG), World Bank, National Statistical Office of Mongolia. Mongolia Population and Housing Census 1979
22. Dugee O, Khor GL, Lye M-S, Luvsannyam L, Janchiv O, Jamyan B, Esa N. Association of major dietary patterns with obesity risk among Mongolian men and women. Asia Pac J Clin Nutr. 2009; 18(3): 433-40
23. European Centre for Medium-Range Weather Forecasts (ECMWF). European Reanalysis-5. United Kingdom: European Centre for Medium-Range Weather Forecasts (ECMWF)
24. FAO Supply Utilization Accounts 1961-2013. Personal Correspondence with Dr. Josef Schmidhuber, 2016. [Unpublished]
25. Feenstra, Robert C., Robert Inklaar and Marcel P. Timmer (2015), "The Next Generation of the Penn World Table" American Economic Review, 105(10), 3150-3182, available for download at www.ggdc.net/pwt
26. Food and Agriculture Organization of the United Nations (FAO). FAOSTAT Commodity Balances - Crops Primary Equivalent. Rome, Italy: Food and Agriculture Organization of the United Nations (FAO)
27. Food and Agriculture Organization of the United Nations (FAO). FAOSTAT Food Balance Sheets, October 2014. Rome, Italy: Food and Agriculture Organization of the United Nations (FAO)
28. Food Fortification Initiative (United States), Global Alliance for Improved Nutrition (GAIN), Iodine Global Network (Canada), Micronutrient Forum (Canada). Global Fortification Data Exchange: Legislation and Standards, Year When Food Fortification Mandated, and Count of Nutrients In Fortification Standards
29. Foster PJ, Baasanhu J, Alsbirk PH, Munkhbayar D, Uranchimeg D, Johnson GJ. Glaucoma in Mongolia. A population-based survey in Hövsgöl province, northern Mongolia. Arch Ophthalmol. 1996; 114(10): 1235-41
30. Fuse Y, Igari T, Yamada C, Sakano S, Ito H, Umenai T, Irie M. Epidemiological survey of thyroid volume and iodine intake in schoolchildren, postpartum women and neonates living in Ulaan Baatar. Clin Endocrinol (Oxf). 2003; 59(3): 298-306 as it appears in World Health Organization (WHO). WHO Global Database on Iodine Deficiency. Geneva, Switzerland: World Health Organization (WHO)
31. Gallup. Mongolia World Poll 2011
32. Gallup. Mongolia World Poll 2012
33. Global Burden of Disease 2010 Expert Group Working Document on Sense and Hearing Disorders
34. Global Burden of Disease Collaborative Network. Global Burden of Disease Study 2016 (GBD 2016) Covariates 1980-2016. Seattle, United States: Institute for Health Metrics and Evaluation (IHME), 2017
35. Global Burden of Disease Collaborative Network. Global Burden of Disease Study 2017 (GBD 2017) Cause-Specific Mortality 1980-2017. Seattle, United States: Institute for Health Metrics and Evaluation (IHME), 2018
36. Global Burden of Disease Collaborative Network. Global Health Spending 1995-2017. Seattle, United States of America: Institute for Health Metrics and Evaluation (IHME), 2020
37. Government of Mongolia, National Center for Communicable Diseases (Mongolia), Research Institute of Tuberculosis/Japan Anti-Tuberculosis Association (RIT/JATA), World Health Organization (WHO). Mongolia National Tuberculosis Prevalence Survey 2014-2015
38. Government of Mongolia, National Statistical Office of Mongolia, United Nations Children's Fund (UNICEF), United Nations Population Fund (UNFPA). Mongolia Multiple Indicator Cluster Survey 2013. New York, United States of America: United Nations Children's Fund (UNICEF), 2016
39. International Centre for Eye Health (ICEH). Mongolia Rapid Assessment of Avoidable Blindness Survey 2013. Grootebroek, Netherlands: RAAB Repository, 2014
40. International Fuel Quality Center (IFQC). Overview of Leaded Gasoline and Sulfur Levels in Gasoline and Diesel . 2002
41. International Labour Organization (ILO), National Statistical Office of Mongolia. Mongolia Labor Force Survey 2008-2009. Ulaanbaatar, Mongolia: National Statistical Office of Mongolia
42. International Labour Organization (ILO), National Statistical Office of Mongolia. Mongolia Labor Force Survey 2010. Ulaanbaatar, Mongolia: National Statistical Office of Mongolia
43. International Labour Organization (ILO), National Statistical Office of Mongolia. Mongolia Labor Force Survey 2012. Ulaanbaatar, Mongolia: National Statistical Office of Mongolia
44. International Labour Organization (ILO), National Statistical Office of Mongolia. Mongolia Labor Force Survey 2013. Ulaanbaatar, Mongolia: National Statistical Office of Mongolia
45. International Labour Organization (ILO), National Statistical Office of Mongolia. Mongolia Labor Force Survey 2014. Ulaanbaatar, Mongolia: National Statistical Office of Mongolia
46. International Labour Organization (ILO), National Statistical Office of Mongolia. Mongolia Labor Force Survey with Child Labor Survey 2002-2003. Ulaanbaatar, Mongolia: National Statistical Office of Mongolia
47. International Labour Organization (ILO), National Statistical Office of Mongolia. Mongolia Labor Force Survey with Child Labor Survey 2006-2007. Ulaanbaatar, Mongolia: National Statistical Office of Mongolia
48. International Labour Organization (ILO), National Statistical Office of Mongolia. Mongolia Labor Force Survey with Informal Sector Survey 2007-2008. Ulaanbaatar, Mongolia: National Statistical Office of Mongolia
49. International Labour Organization (ILO). International Labour Organization Database (ILOSTAT) - Employment by Sex and Economic Activity. International Labour Organization (ILO)
50. International Labour Organization (ILO). International Labour Organization Database (ILOSTAT) - Employment by Sex and Occupation. International Labour Organization (ILO)
51. International Labour Organization (ILO). International Labour Organization Database (ILOSTAT) - Employment to Population Ratio by Sex and Age. International Labour Organization (ILO)
52. International Monetary Fund (IMF). World Economic Outlook Database. Washington, D.C., United States of America: International Monetary Fund (IMF)
53. International Road Federation. World Road Statistics 1963-1999. Geneva, Switzerland: International Road Federation
54. International Road Federation. World Road Statistics 2009 Database and World Road Statistics 2007
55. International Road Federation. World Road Statistics 2015. Geneva, Switzerland: International Road Federation, 2015
56. International Road Federation. World Road Statistics 2018. Geneva, Switzerland: International Road Federation, 2018
57. International Road Federation. World Road Statistics 2019. Geneva, Switzerland: International Road Federation, 2019
58. International Society of Nephrology (ISN). Mongolia - Ulaanbaatar International Society of Nephrology Kidney Disease Data Center Chronic Kidney Disease and Cardiovascular Risk Survey 2007
59. Joint United Nations Program on HIV/AIDS (UNAIDS), United Nations Children's Fund (UNICEF), World Health Organization (WHO). Mongolia Global AIDS Response Progress Reporting (GARPR) System - Antenatal Care Attendees Positive for Syphilis
60. Kachondham Y, Dhanamitta S, Oyunbileg M, Brown L. Child health and nutritional status in Ulaanbaatar, Mongolia: a preliminary assessment. Asia Pac J Public Health. 1992; 6(4): 226-32 as it appears in World Health Organization (WHO). WHO Global Database on Child Growth and Malnutrition - Historical. Geneva, Switzerland: World Health Organization (WHO)
61. Kirillova NV, Fedosova EA, Naranbat N, Oyuntuya T, Buyankhishig B, Enkhsaikhan D, Demkin VV, Nymadawa P. Structure of the M-tuberculosis Population in Mongolia According to the Results of Genotyping of Large-sequence Polymorphisms. Mol Genet Microbiol Virol. 2011; 26(4): 141-5
62. Liu Z, Huang Y, Chen X, Cheng H, Luo X. A cross-sectional study of 4 mental disorders in Chifeng City of Inner Mongolia Autonomous Region. Chin Ment Health J. 2015; 29(9): 678–84
63. London School of Hygiene and Tropical Medicine. Global Atlas of Helminth Infections - Soil Transmitted Helminths. London, United Kingdom: London School of Hygiene and Tropical Medicine
64. Maddison Project Database, version 2018. Bolt, Jutta, Robert Inklaar, Herman de Jong and Jan Luiten van Zanden (2018), "Rebasing 'Maddison': new income comparisons and the shape of long-run economic development", Maddison Project Working paper 10
65. Malaria Atlas Project. Sickle Haemoglobin (Hbs) Allele Frequency Layer. Oxford, United Kingdom: Malaria Atlas Project, 2013
66. Max Planck Institute for Demographic Research, Vienna Institute of Demography. Human Fertility Collection - ASFR, original age scale. Rostock, Germany: Max Planck Institute for Demographic Research
67. Mendsaikhan J, Watt JP, Mansoor O, Suvdmaa N, Edmond K, Litt DJ, Nymadawa P, Yang Baoping, Altantsetseg D, Slack M. Childhood Bacterial Meningitis in Ulaanbaatar, Mongolia, 2002-2004. Clin Infect Dis. 2009; 48 Suppl 2(Suppl 2): S141-6
68. Ministry of Health (Mongolia), National Statistical Office of Mongolia, United Nations Population Fund (UNFPA). Mongolia Reproductive Health Survey 2003. Ulaanbaatar, Mongolia: National Statistical Office of Mongolia, 2004
69. Ministry of Health (Mongolia), National Statistical Office of Mongolia. Mongolia Reproductive Health Survey 2008. Ulaanbaatar, Mongolia: National Statistical Office of Mongolia
70. Ministry of Health (Mongolia). Mongolia Health Indicators 2001. Ulaanbaatar, Mongolia: Ministry of Health (Mongolia)
71. Ministry of Health (Mongolia). Mongolia Health Indicators 2003. Ulaanbaatar, Mongolia: Ministry of Health (Mongolia)
72. Ministry of Health (Mongolia). Mongolia Health Indicators 2006. Ulaanbaatar, Mongolia: Ministry of Health (Mongolia)
73. Ministry of Health (Mongolia). Mongolia Health Indicators 2007. Ulaanbaatar, Mongolia: Ministry of Health (Mongolia)
74. Ministry of Health (Mongolia). Mongolia Sexually Transmitted Infections among Pregnant Women Attending Antenatal Clinics 2008
75. Ministry of Health and Social Welfare (Mongolia), National Statistical Office of Mongolia, United Nations Statistics Division (UNSD). Mongolia Reproductive Health Survey 1998
76. Ministry of Immigration and Population (Myanmar), United Nations Population Fund (UNFPA). Myanmar Fertility and Reproductive Health Survey 2006-2007
77. Mongolia - Ulaanbaatar Air Quality Monitoring Network 2011 as it appears in Shaddick G, Thomas ML. Particulate Matter 2.5 and 10 Surface Monitor Station Expanded Database 2008-2017. [Unpublished]
78. Mongolia - Ulaanbaatar Air Quality Monitoring Network 2012 as it appears in Shaddick G, Thomas ML. Particulate Matter 2.5 and 10 Surface Monitor Station Expanded Database 2008-2017. [Unpublished]
79. Mongolia - Ulaanbaatar Air Quality Monitoring Network 2013 as it appears in Shaddick G, Thomas ML. Particulate Matter 2.5 and 10 Surface Monitor Station Expanded Database 2008-2017. [Unpublished]
80. Mongolia - Ulaanbaatar Air Quality Monitoring Network 2014 as it appears in Shaddick G, Thomas ML. Particulate Matter 2.5 and 10 Surface Monitor Station Expanded Database 2008-2017. [Unpublished]
81. Mongolia - Ulaanbaatar Air Quality Monitoring Network 2015 as it appears in Shaddick G, Thomas ML. Particulate Matter 2.5 and 10 Surface Monitor Station Expanded Database 2008-2017. [Unpublished]
82. Mongolia - Ulaanbaatar AirNow Air Quality Data 2016 as it appears in Shaddick G, Thomas ML. Particulate Matter 2.5 and 10 Surface Monitor Station Expanded Database 2008-2017. [Unpublished]
83. Mongolia Child Nutrition Survey 1992 as it appears in World Health Organization (WHO). WHO Global Database on Child Growth and Malnutrition. Geneva, Switzerland: World Health Organization (WHO)
84. Mongolia Chronic Kidney Disease Statistics 1961-2011, as provided by the Global Burden of Disease 2010 Chronic Kidney Disease Expert Group. [Unpublished]
85. Mongolia Iodine Deficiency Disorder Survey 1993 as it appears in World Health Organization (WHO). WHO Global Database on Iodine Deficiency. Geneva, Switzerland: World Health Organization (WHO)
86. Mongolia Iodine Deficiency Disorder Survey 2001 as it appears in World Health Organization (WHO). WHO Global Database on Iodine Deficiency. Geneva, Switzerland: World Health Organization (WHO)
87. Mongolia National Nutrition Survey 2010 as it appears in Petry N, Olofin I, Hurrell RF, Boy E, Wirth JP, Moursi M, Angel MD, Rohner F. The Proportion of Anemia Associated with Iron Deficiency in Low, Medium, and High Human Development Index Countries: A Systematic Analysis of National Surveys. Nutrients. 2016; 8(11)
88. Mongolia Population and Housing Census 1956 as it appears in United Nations Statistics Division (UNSD). United Nations Demographic Yearbook. New York City, United States: United Nations Statistics Division (UNSD)
89. Mongolia Population and Housing Census 1989 as it appears in United Nations Statistics Division (UNSD). United Nations Demographic Yearbook. New York City, United States: United Nations Statistics Division (UNSD)
90. Mongolia Population and Housing Census 2000 as it appears in United Nations Statistics Division (UNSD). United Nations Demographic Yearbook. New York City, United States: United Nations Statistics Division (UNSD)
91. Mongolia Population and Housing Census 2010 as it appears in United Nations Statistics Division (UNSD). United Nations Demographic Yearbook. New York City, United States: United Nations Statistics Division (UNSD)
92. Mongolia Vital Registration - Deaths 1994 ICD9 as it appears in World Health Organization (WHO). WHO Mortality Database Version September 2016. Geneva, Switzerland: World Health Organization (WHO), 2016
93. Mongolia Vital Registration - Deaths 2016 ICD10 as it appears in World Health Organization (WHO). WHO Mortality Database Version April 2018. Geneva, Switzerland: World Health Organization (WHO), 2018
94. Mongolia Vital Registration Death Data 2009 as it appears in United Nations Statistics Division (UNSD). United Nations Demographic Yearbook 2009-2010. New York, United States: United Nations (UN), 2011
95. Mongolia Vital Registration Death Data 1965 as it appears in United Nations Statistics Division (UNSD). United Nations Demographic Yearbook. New York City, United States: United Nations Statistics Division (UNSD)
96. Mongolia Vital Registration Death Data 1970 as it appears in United Nations Statistics Division (UNSD). United Nations Demographic Yearbook. New York City, United States: United Nations Statistics Division (UNSD)
97. Mongolia Vital Registration Death Data 1975 as it appears in United Nations Statistics Division (UNSD). United Nations Demographic Yearbook. New York City, United States: United Nations Statistics Division (UNSD)
98. Mongolia Vital Registration Death Data 1980 as it appears in United Nations Statistics Division (UNSD). United Nations Demographic Yearbook. New York City, United States: United Nations Statistics Division (UNSD)
99. Mongolia Vital Registration Death Data 1985 as it appears in United Nations Statistics Division (UNSD). United Nations Demographic Yearbook. New York City, United States: United Nations Statistics Division (UNSD)
100. Mongolia Vital Registration Death Data 1987 as it appears in United Nations Statistics Division (UNSD). United Nations Demographic Yearbook - Historical Supplement 1997. New York, United States of America: United Nations (UN)
101. Mongolia Vital Registration Death Data 1988 as it appears in United Nations Statistics Division (UNSD). United Nations Demographic Yearbook - Historical Supplement 1997. New York, United States of America: United Nations (UN)
102. Mongolia Vital Registration Death Data 1989 as it appears in United Nations Statistics Division (UNSD). United Nations Demographic Yearbook - Historical Supplement 1997. New York, United States of America: United Nations (UN)
103. Mongolia Vital Registration Death Data 1990 as it appears in United Nations Statistics Division (UNSD). United Nations Demographic Yearbook. New York City, United States: United Nations Statistics Division (UNSD)
104. Mongolia Vital Registration Death Data 1995 as it appears in United Nations Statistics Division (UNSD). United Nations Demographic Yearbook. New York City, United States: United Nations Statistics Division (UNSD)
105. Mongolia Vital Registration Death Data 1996 as it appears in United Nations Statistics Division (UNSD). United Nations Demographic Yearbook. New York City, United States: United Nations Statistics Division (UNSD)
106. Mongolia Vital Registration Death Data 1997 as it appears in United Nations Statistics Division (UNSD). United Nations Demographic Yearbook. New York City, United States: United Nations Statistics Division (UNSD)
107. Mongolia Vital Registration Death Data 1998 as it appears in United Nations Statistics Division (UNSD). United Nations Demographic Yearbook 2006. New York, United States: United Nations (UN), 2008
108. Mongolia Vital Registration Death Data 2000 as it appears in United Nations Statistics Division (UNSD). United Nations Demographic Yearbook 2001. New York, United States: United Nations (UN), 2003
109. Mongolia Vital Registration Death Data 2001 as it appears in United Nations Statistics Division (UNSD). United Nations Demographic Yearbook. New York City, United States: United Nations Statistics Division (UNSD)
110. Mongolia Vital Registration Death Data 2006 as it appears in United Nations Statistics Division (UNSD). United Nations Demographic Yearbook 2006. New York, United States: United Nations (UN), 2008
111. Mongolia Vital Registration Death Data 2007 as it appears in United Nations Statistics Division (UNSD). United Nations Demographic Yearbook 2007. New York, United States: United Nations (UN), 2009
112. Mongolia Vital Registration Death Data 2008 as it appears in United Nations Statistics Division (UNSD). United Nations Demographic Yearbook 2008. New York, United States: United Nations (UN), 2010
113. Mongolia Vital Registration Death Data 2013 as it appears in United Nations Statistics Division (UNSD). United Nations Demographic Yearbook 2013. New York, United States: United Nations (UN), 2014
114. Mongolia Vital Registration Death Data 2014 as it appears in United Nations Statistics Division (UNSD). United Nations Demographic Yearbook 2014. New York, United States: United Nations (UN), 2015

115 Mongolia Vital Registration Live Births Data 1955 as it appears in United Nations Statistics Division (UNSD). United Nations Demographic Yearbook - Historical Supplement 1997. New York, United States of America: United Nations (UN)
116 Mongolia Vital Registration Live Births Data 1956 as it appears in United Nations Statistics Division (UNSD). United Nations Demographic Yearbook - Historical Supplement 1997. New York, United States of America: United Nations (UN)
117 Mongolia Vital Registration Live Births Data 1957 as it appears in United Nations Statistics Division (UNSD). United Nations Demographic Yearbook - Historical Supplement 1997. New York, United States of America: United Nations (UN)
118 Mongolia Vital Registration Live Births Data 1958 as it appears in United Nations Statistics Division (UNSD). United Nations Demographic Yearbook - Historical Supplement 1997. New York, United States of America: United Nations (UN)
119 Mongolia Vital Registration Live Births Data 1960 as it appears in United Nations Statistics Division (UNSD). United Nations Demographic Yearbook - Historical Supplement 1997. New York, United States of America: United Nations (UN)
120 Mongolia Vital Registration Live Births Data 1965 as it appears in United Nations Statistics Division (UNSD). United Nations Demographic Yearbook - Historical Supplement 1997. New York, United States of America: United Nations (UN)
121 Mongolia Vital Registration Live Births Data 1978 as it appears in United Nations Statistics Division (UNSD). United Nations Demographic Yearbook - Historical Supplement 1997. New York, United States of America: United Nations (UN)
122 Mongolia Vital Registration Live Births Data 1979 as it appears in United Nations Statistics Division (UNSD). United Nations Demographic Yearbook - Historical Supplement 1997. New York, United States of America: United Nations (UN)
123 Mongolia Vital Registration Live Births Data 1981 as it appears in United Nations Statistics Division (UNSD). United Nations Demographic Yearbook - Historical Supplement 1997. New York, United States of America: United Nations (UN)
124 Mongolia Vital Registration Live Births Data 1983 as it appears in United Nations Statistics Division (UNSD). United Nations Demographic Yearbook - Historical Supplement 1997. New York, United States of America: United Nations (UN)
125 Mongolia Vital Registration Live Births Data 1984 as it appears in United Nations Statistics Division (UNSD). United Nations Demographic Yearbook - Historical Supplement 1997. New York, United States of America: United Nations (UN)
126 Mongolia Vital Registration Live Births Data 1986 as it appears in United Nations Statistics Division (UNSD). United Nations Demographic Yearbook - Historical Supplement 1997. New York, United States of America: United Nations (UN)
127 Mongolia Vital Registration Live Births Data 1987 as it appears in United Nations Statistics Division (UNSD). United Nations Demographic Yearbook - Historical Supplement 1997. New York, United States of America: United Nations (UN)
128 Mongolia Vital Registration Live Births Data 1988 as it appears in United Nations Statistics Division (UNSD). United Nations Demographic Yearbook - Historical Supplement 1997. New York, United States of America: United Nations (UN)
129 Mongolia Vital Registration Live Births Data 1989 as it appears in United Nations Statistics Division (UNSD). United Nations Demographic Yearbook - Historical Supplement 1997. New York, United States of America: United Nations (UN)
130 Mongolia World Poll 2007 as it appears in Gallup. Gallup Analytics - World Poll Negative Experience Index. Washington, D.C., United States: Gallup, 2019
131 Mongolia World Poll 2008 as it appears in Gallup. Gallup Analytics - World Poll Negative Experience Index. Washington, D.C., United States: Gallup, 2019
132 Mongolia World Poll 2010 as it appears in Gallup. Gallup Analytics - World Poll Negative Experience Index. Washington, D.C., United States: Gallup, 2019
133 Mongolia World Poll 2013 as it appears in Gallup. Gallup Analytics - World Poll Negative Experience Index. Washington, D.C., United States: Gallup, 2019
134 Mongolia World Poll 2014 as it appears in Gallup. Gallup Analytics - World Poll Negative Experience Index. Washington, D.C., United States: Gallup, 2019
135 Mongolia World Poll 2015 as it appears in Gallup. Gallup Analytics - World Poll Negative Experience Index. Washington, D.C., United States: Gallup, 2019
136 Mongolia World Poll 2016 as it appears in Gallup. Gallup Analytics - World Poll Negative Experience Index. Washington, D.C., United States: Gallup, 2019
137 Mongolia World Poll 2017 as it appears in Gallup. Gallup Analytics - World Poll Negative Experience Index. Washington, D.C., United States: Gallup, 2019
138 Mongolia World Poll 2018 as it appears in Gallup. Gallup Analytics - World Poll Negative Experience Index. Washington, D.C., United States: Gallup, 2019
139 Morocco National Survey of Iron Deficiency, Use of Iodized Salt, and Vitamin A Supplementation 2000 as it appears in World Health Organization (WHO). WHO Global Database on Anemia, Nutrition Landscape Information System. Geneva, Switzerland: World Health Organization (WHO)
140 National Statistical Office of Mongolia, Minnesota Population Center. Mongolia Population and Housing Census 1989 from the Integrated Public Use Microdata Series, International: [Machine-readable database]. Minneapolis: University of Minnesota
141 National Statistical Office of Mongolia, Minnesota Population Center. Mongolia Population and Housing Census 2000 from the Integrated Public Use Microdata Series, International: [Machine-readable database]. Minneapolis: University of Minnesota
142 National Statistical Office of Mongolia, Statistics Department of Khuvsgul Aimag (Mongolia), United Nations Children's Fund (UNICEF). Mongolia - Khuvsgul Multiple Indicator Cluster Survey 2012. New York, United States of America: United Nations Children's Fund (UNICEF), 2015
143 National Statistical Office of Mongolia, Statistics Department, Governor’s Office of the Nalaikh (Mongolia), United Nations Children's Fund (UNICEF). Mongolia - Nalaikh District Multiple Cluster Indicator Survey 2012. New York, United States of America: United Nations Children's Fund (UNICEF), 2017
144 National Statistical Office of Mongolia, United Nations Children's Fund (UNICEF). Mongolia - Khuvsgul Multiple Indicator Cluster Survey 2016. New York, United States of America: United Nations Children's Fund (UNICEF), 2018
145 National Statistical Office of Mongolia, United Nations Children's Fund (UNICEF). Mongolia - Nalaikh District Multiple Indicator Cluster Survey 2016. New York, United States of America: United Nations Children's Fund (UNICEF), 2018
146 National Statistical Office of Mongolia, United Nations Children's Fund (UNICEF). Mongolia Multiple Indicator Cluster Survey 1996
147 National Statistical Office of Mongolia, United Nations Children's Fund (UNICEF). Mongolia Multiple Indicator Cluster Survey 2005. New York, United States of America: United Nations Children's Fund (UNICEF)
148 National Statistical Office of Mongolia, United Nations Children's Fund (UNICEF). Mongolia Multiple Indicator Cluster Survey 2010. New York, United States of America: United Nations Children's Fund (UNICEF), 2013
149 National Statistical Office of Mongolia. Mongolia Birth Rates by Age of Mother. Ulaanbaatar, Mongolia: National Statistical Office of Mongolia
150 National Statistical Office of Mongolia. Mongolia Multiple Indicator Cluster Survey 2000. New York, United States of America: United Nations Children's Fund (UNICEF)
151 National Statistical Office of Mongolia. Mongolia Statistical Yearbook 2001. Ulaanbaatar, Mongolia: National Statistical Office of Mongolia, 2002
152 National Statistical Office of Mongolia. Mongolia Statistical Yearbook 2002. Ulaanbaatar, Mongolia: National Statistical Office of Mongolia
153 National Statistical Office of Mongolia. Mongolia Statistical Yearbook 2003
154 National Statistical Office of Mongolia. Mongolia Statistical Yearbook 2004. Ulaanbaatar, Mongolia: National Statistical Office of Mongolia, 2005
155 National Statistical Office of Mongolia. Mongolia Statistical Yearbook 2005. Ulaanbaatar, Mongolia: National Statistical Office of Mongolia, 2006
156 Nyambat B, Gantuya S, Batuwanthudawe R, Wijesinghe PR, Abeysinghe N, Galagoda G, Kirkwood C, Bogdanovic-Sakran N, Kang JO, Kilgore PE. Epidemiology of rotavirus diarrhea in Mongolia and Sri Lanka, March 2005-February 2007. J Infect Dis. 2009; 200(Suppl 1): 160-166
157 Orsoo O, Saw YM, Sereenen E, Yadamsuren B, Byambaa A, Kariya T, Yamamoto E, Hamajima N. Epidemiological characteristics and trends of a Nationwide measles outbreak in Mongolia, 2015-2016. BMC Public Health. 2019; 19(1): 201
158 Oyunsuren T, Kurbanov F, Tanaka Y, Elkady A, Sanduijav R, Khajidsuren O, Dagvadorj B, Mizokami M. High frequency of hepatocellular carcinoma in Mongolia; association with mono-, or co-infection with hepatitis C, B, and delta viruses. J Med Virol. 2006; 78(12): 1688-95
159 Pew Research Center. The Future of the Global Muslim Population. Washington, DC, United States: Pew Research Center, 2011
160 Popova S, Lange S, Probst C, Gmel G, Rehm J, Centre for Addiction and Mental Health (Canada). Estimation of national, regional and global prevalence of alcohol use during pregnancy and fetal alcohol syndrome: a systematic review and meta-analysis. Lancet Glob Health. [Forthcoming]
161 Public Health Institute, Ministry of Health (Mongolia), United Nations Children's Fund (UNICEF). Mongolia National Nutrition Survey 2004
162 Pullan RL, Smith JL, Jasrasaria R, Brooker SJ. Global numbers of infection and disease burden of soil transmitted helminth infections in 2010 [Unpublished data]. Parasit Vectors. 2014; 7(37). [Unpublished data as provided by the Global Burden of Disease 2010 soil transmitted helminths expert group]
163 Samdan A, Ganbold S, Guntev O, Orosoo S, Javzandorj N, Gongor A, Enkhtuvshin A, Demberelsuren S, Abdul W, Jee Y, Grabovac V, Kirkwood C, Fox K, Nyambat B. Hospital-based surveillance for rotavirus diarrhea in Ulaanbaatar, Mongolia, April 2009 through March 2016. Vaccine. 2018
164 Scott S, Altanseseg D, Sodbayer D, Nymadawa P, Bulgan D, Mendsaikhan J, Watt JP, Slack MP, Carvalho MG, Hajjeh R, Edmond KM. Impact of Haemophilus influenzae Type b conjugate vaccine in Mongolia: prospective population-based surveillance, 2002-2010. J Pediatr. 2013; 163(1 Suppl): S8-S11
165 Shaddick G, Thomas ML. Particulate Matter 2.5 and 10 Surface Monitor Station Expanded Database 2008-2017. [Unpublished]
166 Suvd J, Gerel B, Otgooloi H, Purevsuren D, Zolzaya H, Roglic G, King H. Glucose intolerance and associated factors in Mongolia: results of a national survey. Diabet Med. 2002; 19(6): 502–8
167 Takahashi M, Nishizawa T, Gotanda Y, Tsuda F, Komatsu F, Kawabata T, Hasegawa K, Altankhuu M, Chimedregzen U, Narantuya L, Hoshino H, Hino K, Kagawa Y, Okamoto H. High prevalence of antibodies to hepatitis A and E viruses and viremia of hepatitis B, C, and D viruses among apparently healthy populations in Mongolia. Clin Diagn Lab Immunol. 2004; 11(2): 392-8
168 Tsatsralt-Od B, Takahashi M, Endo K, Agiimaa D, Buyankhuu O, Okamoto H. Comparison of hepatitis A and E virus infections among healthy children in Mongolia: evidence for infection with a subgenotype IA HAV in children. J Med Virol. 2007; 79(1): 18-25
169 Tsatsralt-Od B, Takahashi M, Nishizawa T, Endo K, Inoue J, Okamoto H. High prevalence of dual or triple infection of hepatitis B, C, and delta viruses among patients with chronic liver disease in Mongolia. J Med Virol. 2005; 77(4): 491-9
170 Tserenpuntsag B, Ouynbileg L, Nelson K, McNutt L-A. Prevalence of infectious diseases among Mongolian blood donors. J Infect Dev Ctries. 2008; 2(1): 73–5
171 United Nations Children's Fund (UNICEF), World Health Organization (WHO). WHO and UNICEF Reported Disease Incidence Time Series. Geneva, Switzerland: World Health Organization (WHO)
172 United Nations Children's Fund (UNICEF), World Health Organization (WHO). WHO and UNICEF Reported Official Target Population, Number of Doses Administered and Official Coverage 1966-2018. Geneva, Switzerland: World Health Organization (WHO), 2019
173 United Nations Children's Fund (UNICEF). UNICEF Maternal and Newborn Health Coverage Database as of November 2019. New York, United States of America: United Nations Children's Fund (UNICEF), 2019
174 United Nations Children's Fund (UNICEF). UNICEF State of the World's Children 2017. New York, United States: United Nations Children's Fund (UNICEF), 2017
175 United Nations Environment Programme. Leaded Petrol Phase-out: Global Status, March 2018. Nairobi, Kenya: United Nations Environment Programme
176 United Nations Office on Drugs and Crime (UNODC). United Nations Office on Drugs and Crime Global Study on Homicide 2011. Vienna, Austria: United Nations Office on Drugs and Crime (UNODC), 2011
177 United Nations Office on Drugs and Crime (UNODC). World Drug Report 2012. Vienna, Austria: United Nations Office on Drugs and Crime (UNODC), 2012
178 United Nations Population Division. Abortion Policies: A Global Review. New York, United States: United Nations (UN), 2002
179 United Nations Population Division. World Abortion Policies 2007. New York, United States: United Nations (UN), 2007
180 United Nations Population Division. World Abortion Policies 2013. New York, United States: United Nations (UN), 2013
181 United Nations Statistics Division (UNSD). National Accounts Main Aggregates Database. New York City, United States of America: United Nations Statistics Division (UNSD)
182 United Nations Statistics Division (UNSD). United Nations Demographic Yearbook - Historical Supplement 1997. New York, United States of America: United Nations (UN)
183 United Nations Statistics Division (UNSD). United Nations Demographic Yearbook 2001. New York, United States: United Nations (UN), 2003
184 United Nations Statistics Division (UNSD). United Nations Demographic Yearbook 2002. New York, United States: United Nations (UN), 2005
185 United Nations Statistics Division (UNSD). United Nations Demographic Yearbook 2004. New York, United States: United Nations (UN), 2007
186 United Nations Statistics Division (UNSD). United Nations Demographic Yearbook 2006. New York, United States: United Nations (UN), 2008
187 United Nations Statistics Division (UNSD). United Nations Demographic Yearbook 2007. New York, United States: United Nations (UN), 2009
188 United Nations Statistics Division (UNSD). United Nations Demographic Yearbook 2009-2010. New York, United States: United Nations (UN), 2011
189 United Nations Statistics Division (UNSD). United Nations Demographic Yearbook 2013. New York, United States: United Nations (UN), 2014
190 United Nations Statistics Division (UNSD). United Nations Demographic Yearbook 2014. New York, United States: United Nations (UN), 2015
191 United Nations Statistics Division (UNSD). United Nations Demographic Yearbook 2015. New York, United States of America: United Nations (UN), 2016
192 United Nations Statistics Division (UNSD). United Nations Demographic Yearbook 2016. New York, United States of America: United Nations (UN), 2017
193 United Nations Statistics Division (UNSD). United Nations Demographic Yearbook. New York City, United States: United Nations Statistics Division (UNSD)
194 Uuriintuya M, Ulziibayar D, Bayarmaa D. Epilepsy in Mongolia. Neurology Asia. 2007; 12: 61-63
195 Wang Y, Peng R, Ma R. Epidemiological investigation of osteoarthritis in middle-aged mongolian and senior residents of the inner mongolia autonomous region. Iran Red Crescent Med J. 2013; 15(10): e8303
196 World Bank. World Development Indicators - Gross Domestic Product (GDP). Washington DC, United States of America: World Bank
197 World Bank. World Development Indicators - Hospital Beds (per 1,000 People). Washington DC, United States: World Bank
198 World Bank. World Development Indicators - Vitamin A Supplementation Coverage Rate. Washington DC, United States: World Bank
199 World Health Organization (WHO), Ministry of Health (Mongolia), National Medical Research Institute (Mongolia), Health Sciences University (Mongolia), National Oncology Center of Mongolia (Mongolia). Mongolia STEPS Noncommunicable Disease Risk Factors Survey 2005
200 World Health Organization (WHO), Ministry of Health (Mongolia), National Medical Research Institute (Mongolia), Health Sciences University (Mongolia), National Oncology Center of Mongolia (Mongolia). Mongolia STEPS Noncommunicable Disease Risk Factors Survey 2009
201 World Health Organization (WHO). Country Mortality Data 1980-1999
202 World Health Organization (WHO). Global leprosy situation, 2005. Wkly Epidemiol Rec. 2005: 80(34): 289-95
203 World Health Organization (WHO). Global leprosy situation, 2006. Wkly Epidemiol Rec. 2006: 81(32): 309-16
204 World Health Organization (WHO). Global leprosy situation, 2007. Wkly Epidemiol Rec. 2007: 82(25): 225-32
205 World Health Organization (WHO). Global leprosy situation, 2009. Wkly Epidemiol Rec. 2009: 84(33): 333-40
206 World Health Organization (WHO). Global leprosy situation, 2010. Wkly Epidemiol Rec. 2010: 85(35): 337-48
207 World Health Organization (WHO). Global leprosy situation, beginning of 2008. Wkly Epidemiol Rec. 2008: 83(33): 293-300
208 World Health Organization (WHO). Global leprosy update, 2013; reducing disease burden. Wkly Epidemiol Rec. 2014; 89(36): 389-400
209 World Health Organization (WHO). Global leprosy update, 2014: need for early case detection. Wkly Epidemiol Rec. 2015; 90(36): 461–74
210 World Health Organization (WHO). Global leprosy: update on the 2012 situation. Wkly Epidemiol Rec. 2013: 88(35): 365-79
211 World Health Organization (WHO). Global Status Report on Road Safety 2009. Geneva, Switzerland: World Health Organization (WHO), 2009
212 World Health Organization (WHO). Global Status Report on Road Safety 2013. Geneva, Switzerland: World Health Organization (WHO), 2013
213 World Health Organization (WHO). Global Status Report on Road Safety 2015. Geneva, Switzerland: World Health Organization (WHO), 2015
214 World Health Organization (WHO). Global Status Report on Road Safety 2018. Geneva, Switzerland: World Health Organization (WHO), 2018
215 World Health Organization (WHO). Leprosy update, 2011. Wkly Epidemiol Rec. 2011: 86(36): 389-99
216 World Health Organization (WHO). WHO Distribution of Measles Cases by Country and by Month 2011-2020
217 World Health Organization (WHO). WHO Global Database on Child Growth and Malnutrition - Historical. Geneva, Switzerland: World Health Organization (WHO)
218 World Health Organization (WHO). WHO Global Database on Child Growth and Malnutrition. Geneva, Switzerland: World Health Organization (WHO)
219 World Health Organization (WHO). WHO Global Database on Vitamin A Deficiency. Geneva, Switzerland: World Health Organization (WHO)
220 World Health Organization (WHO). WHO Global Health Expenditure Database - National Health Accounts Indicators. Geneva, Switzerland: World Health Organization (WHO)
221 World Health Organization (WHO). WHO Global Health Observatory - Cholera: Number of Reported Cases by Country. Geneva, Switzerland: World Health Organization (WHO)
222 World Health Organization (WHO). WHO Global Health Observatory - Recorded Alcohol Per Capita Consumption 1960-1979. Geneva, Switzerland: World Health Organization (WHO)
223 World Health Organization (WHO). WHO Global Health Observatory - Recorded Alcohol Per Capita Consumption 1980-1999. Geneva, Switzerland: World Health Organization (WHO)
224 World Health Organization (WHO). WHO Global Health Observatory - Recorded Alcohol Per Capita Consumption 2000-2009 by country. Geneva, Switzerland: World Health Organization (WHO)
225 World Health Organization (WHO). WHO Global Health Observatory (GHO) Data - Hospital Bed Density Data by Country . Geneva, Switzerland: World Health Organization (WHO)
226 World Health Organization (WHO). WHO Global Project on Anti-Tuberculosis Drug Resistance Surveillance Data 1988-2015
227 World Health Organization (WHO). WHO International Travel and Health, Annex 1, Countries With Risk of Yellow Fever Transmission and Countries Requiring Yellow Fever Vaccination 2017 Update. Geneva, Switzerland: World Health Organization (WHO), 2017
228 World Health Organization (WHO). WHO Mortality Database Version April 2018. Geneva, Switzerland: World Health Organization (WHO), 2018
229 World Health Organization (WHO). WHO Mortality Database Version March 2017. Geneva, Switzerland: World Health Organization (WHO), 2017
230 World Health Organization (WHO). WHO Mortality Database Version September 2016. Geneva, Switzerland: World Health Organization (WHO), 2016

231 World Health Organization (WHO). WHO Tuberculosis Case Notifications. Geneva, Switzerland: World Health Organization (WHO)
232 World Health Organization (WHO). Global leprosy update, 2017: reducing the disease burden due to leprosy. Wkly Epidemiol Rec. 2018; 93(35): 445-56
233 World Health Organization Regional Office for the Western Pacific (WPRO-WHO). Number of Reported Cases of Dengue Fever and Dengue Hemorrhagic Fever in the Western Pacific Region, by Country 2000. Manila, Philippines: World Health Organization Regional Office for the Western Pacific (WPRO-WHO)
234 World Health Organization Regional Office for the Western Pacific (WPRO-WHO). Number of Reported Cases of Dengue Fever and Dengue Hemorrhagic Fever in the Western Pacific Region, by Country 2001. Manila, Philippines: World Health Organization Regional Office for the Western Pacific (WPRO-WHO)
235 World Health Organization Regional Office for the Western Pacific (WPRO-WHO). Number of Reported Cases of Dengue Fever and Dengue Hemorrhagic Fever in the Western Pacific Region, by Country 2002. Manila, Philippines: World Health Organization Regional Office for the Western Pacific (WPRO-WHO)
236 World Health Organization Regional Office for the Western Pacific (WPRO-WHO). Number of Reported Cases of Dengue Fever and Dengue Hemorrhagic Fever in the Western Pacific Region, by Country 2003. Manila, Philippines: World Health Organization Regional Office for the Western Pacific (WPRO-WHO)
237 World Health Organization Regional Office for the Western Pacific (WPRO-WHO). Number of Reported Cases of Dengue Fever and Dengue Hemorrhagic Fever in the Western Pacific Region, by Country 2004. Manila, Philippines: World Health Organization Regional Office for the Western Pacific (WPRO-WHO)
238 World Health Organization Regional Office for the Western Pacific (WPRO-WHO). Number of Reported Cases of Dengue Fever and Dengue Hemorrhagic Fever in the Western Pacific Region, by Country 2005. Manila, Philippines: World Health Organization Regional Office for the Western Pacific (WPRO-WHO)
239 World Health Organization Regional Office for the Western Pacific (WPRO-WHO). Number of Reported Cases of Dengue Fever and Dengue Hemorrhagic Fever in the Western Pacific Region, by Country 2006. Manila, Philippines: World Health Organization Regional Office for the Western Pacific (WPRO-WHO)
240 World Health Organization Regional Office for the Western Pacific (WPRO-WHO). Number of Reported Cases of Dengue Fever and Dengue Hemorrhagic Fever in the Western Pacific Region, by Country 2009. Manila, Philippines: World Health Organization Regional Office for the Western Pacific (WPRO-WHO)
241 World Health Organization Regional Office for the Western Pacific (WPRO-WHO). Number of Reported Cases of Dengue Fever and Dengue Hemorrhagic Fever in the Western Pacific Region, by Country 2010. Manila, Philippines: World Health Organization Regional Office for the Western Pacific (WPRO-WHO)
242 World Health Organization Regional Office for the Western Pacific (WPRO-WHO). WPRO-WHO Health Information and Intelligence Platform - Antenatal Care Coverage - At Least 1 Visit. Manila, Philippines: World Health Organization Regional Office for the Western Pacific (WPRO-WHO)
243 Yadamsuren B, Merialdi M, Davaadorj I, Requejo JH, Betrán AP, Ahmad A, Nymadawa P, Erkhembaatar T, Barcelona D, Ba-Thike K, Hagan RJ, Prado R, Wagner W, Khishgee S, Sodnompil T, Tsedmaa B, Jav B, Govind SR, Purevsuren G, Tsevelmaa B, Soyoltuya B, Johnson BR, Fajans P, Van Look PF, Otgonbold A. Tracking Maternal Mortality Declines in Mongolia Between 1992 and 2007: The Importance of Collaboration. Bull World Health Organ. 2010; 88(3): 1992-198
244 Zhang X, Li S, Wu S, Hao X, Guo S, Suzuki K, Yokomichi H, Yamagata Z. Prevalence of birth defects and risk-factor analysis from a population-based survey in Inner Mongolia, China. BMC Pediatr. 2012; 125

Annex 3. Healthy adjusted (HALE) and disability adjusted life expectancy (DALE): Comparison between Mongolia and Central Asian countires

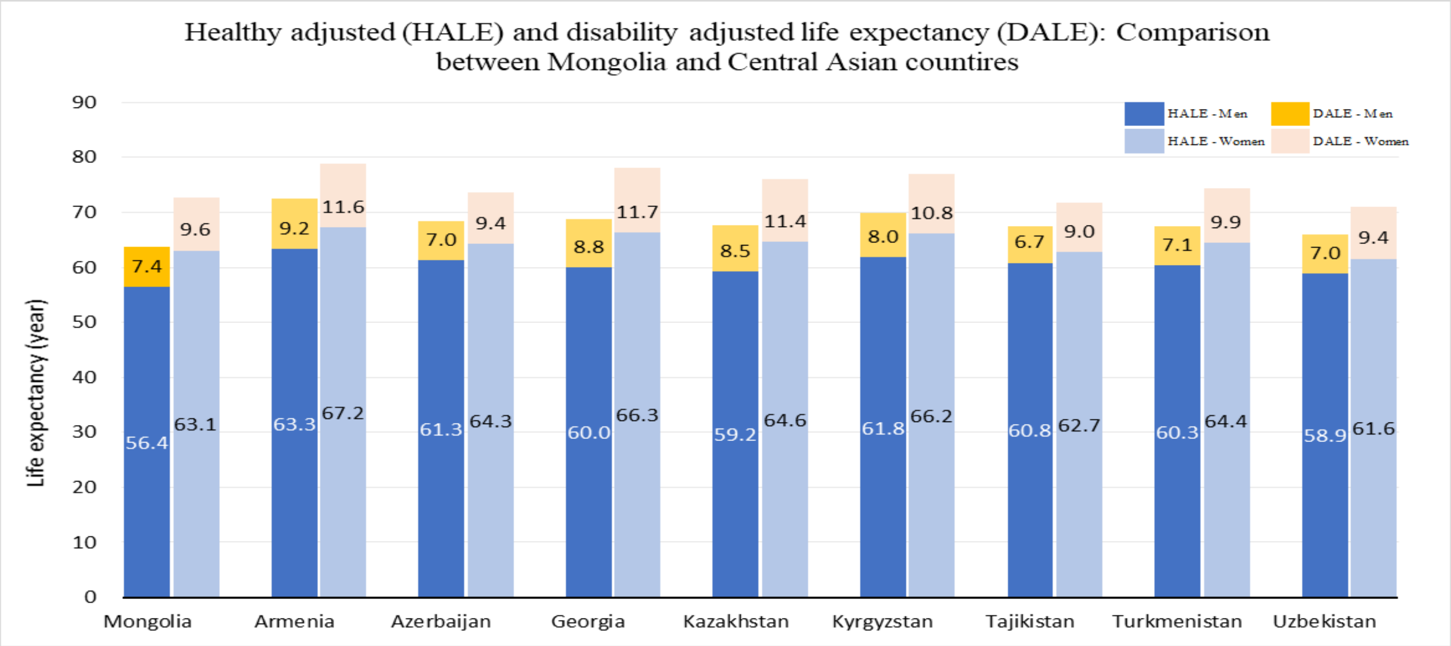

Annex 4. Under 5 Mortality during 1990 and 2019: Comparison between Mongolia and Cenral Asian Countries

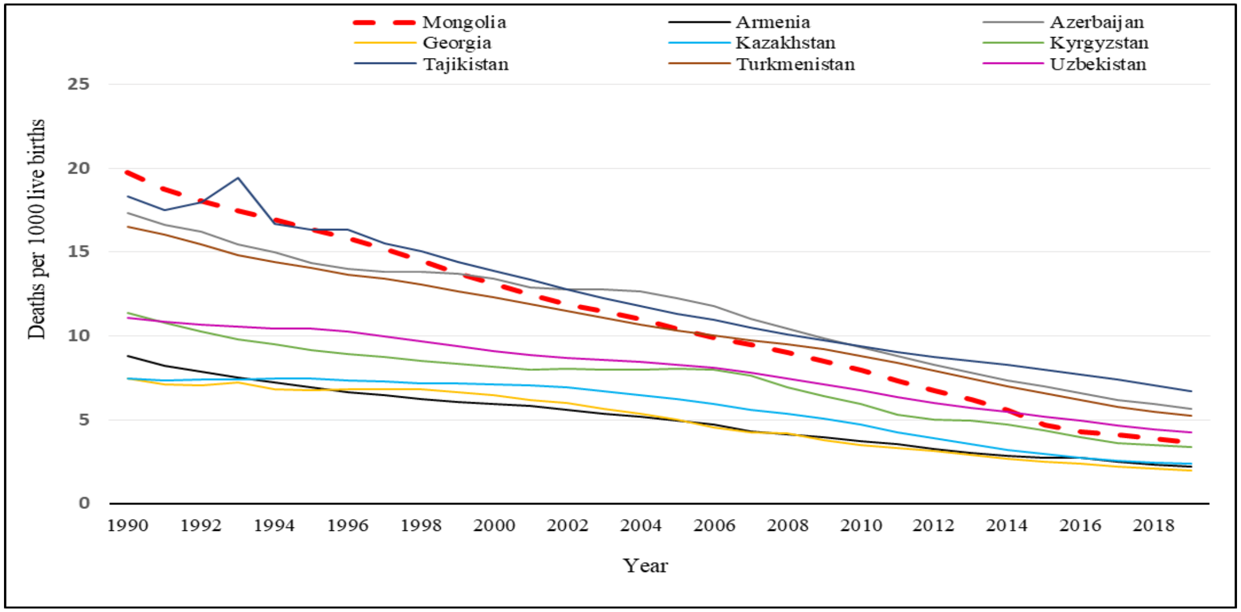

Annex 5. Age-standardized DALY rate attributable to risk factors (GBD Level 2) for men (A) and women (B) in 2019 (Complement to Figure 3)

Risk/Cause	Cardiovascular diseases	Chronic respiratory diseases	Diabetes and kidney diseases	Digestive diseases	Mental disorders	Neoplasms	Neurological disorders	Enteric infections	HIV/AIDS and sexually transmitted infections	Maternal and neonatal disorders	Nutritional deficiencies	Respiratory infections and tuberculosis	Musculoskeletal disorders	Sense organ diseases	Substance use disorders	Transport injuries	Self-harm and interpersonal violence	Unintentional injuries
Unsafe sex	0	0	0	0	0	0	0	0	19.02 (8.8-34.8)	0	0	0	0	0	0	0	0	0
Low physical activity	263.9 (84.5-624.7)	0	14.9 (6.1-28.9)	0	0	7.1 (1.8-15.0)	0	0	0	0	0	0	0	0.0	0	0	0	0
Childhood sexual abuse and bullying	0	0	0	0	42.3 (14.0-86.8)	0	0	0	0	0	0	0	0	0	74.8 (8.8-204.7)	0	0	0
Low bone mineral density	0	0	0	0	0	0	0	0	0	0	0	0	0	0	0	106.8 (74.7-141.3)	9.3 (6.1-13.4)	136.7 (98.8-180.9)
Unsafe water, sanitationand handwashing	0	0	0	0	0	0	0	210 (140.1-292.0)	0	0	0	90 (37.3-153.8)	0	0	0	0	0	0
Other environmental risks	548.0 (237.0-918.3)	0	14.5 (6.4-25.0)	0	16.1 (5.5-30.8)	128.3 (9.1-398.3)	0	0	0	0	0	0	0	0	0	0	0	0
Occupational risks	0	107.5 (82.1-144.9)	0	0	0	143.2 (86.9-222.8)	0	0	0	0	0	0	216.9 (148.8-300.4)	82.9 (5.9-119.4)	0	117.5 (91.5-149.3)	0	165.7 (124.6-217.3)
Drug use	0	0	0	323.4 (185.4-506.3)	0	345.1 (179.1-606.9)	0	0	21.1 (2.9-51.0)	0	0	0	0	0	191.6 (145.0-245.5)	0	24.9 (12.1-43.8)	0
Non-optimal temperature	1407.1 (678.4-2371.8)	102 (58.7-156.4)	69.6 (-20.1-189.4)	0	0	0	0	0	0	0	0	193.0 (39.8-391.7)	0	0	0	0	0	0
High fasting plasma glucose	1249.3 (817.7-1829.0)	0	625.6 (499.4-772.1)	0	0	77.5 (15.7-186.0)	15.8 (2.0-52.6)	0	0	0	0	33.1 (20.2-50.7)	0	2.7 (0.6-6.6)	0	0	0	0
Kidney dysfunction	1456.6 (1027.5-1937.5)	0	679.9 (554.6-829.5)	0	0	0	0	0	0	0	0	0	2.9 (1.7-4.3)	0	0	0	0	0
Child and maternal malnutrition	0	0	0	0	0	0	0	53.1 (22.1-106.7)	0	1310.5 (1026.9-1668)	370.6 (236.2-539.0)	389.8 (244.7-566.4)	0	0	0	0	0	0
High LDL cholesterol	4126.2 (3081.5-5348.8)	0	0	0	0	0	0	0	0	0	0	0	0	0	0	0	0	0
Air pollution	4120.8 (3132.7-5401.1)	184.3 (127.0-260.6)	79.8 (53.8-111.4)	0	0	333.3 (218.4-475.6)	0	1.6 (0.3-4.3)	0	256.8 (61.7-472.3)	0	262.8 (159.3-397.4)	0	15.4 (6.8-25.8)	0	0	0	0
High body-mass index	4050.5 (2394.8-6033.4)	51.6 (26.5-88.0)	415.7 (264.5-581.0)	22.2 (11.1-38.7)	0	842.8 (356.8-1543.3)	38.8 (5.9-107.2)	0	0	0	0	0	77.1 (39.1-132.9)	2.6 (1.0-5.1)	0	0	0	0
Dietary risks	8388.7 (6515.4-10576)	0	209.8 (143.1-286.8)	0	0	553.9 (330.6-956.5)	0	0	0	0	0	0	0	0	0	0	0	0
Tobacco	4918.3 (3858.5-6244.2)	416.9 (335.4-547.4)	96.9 (69.9-127.5)	108.1 (81.6-146.1)	0	2664.3 (2025.4-3446.6)	84.6 (24.7-218.5)	0	0	0	0	476.4 (355.8-616.1)	242.3 (163.2-332.7)	7.8 (5.1-11.4)	0	16.9 (10.7-24.6)	1.6 (0.9-2.4)	25.9 (17.5-37.1)
High systolic blood pressure	9392.5 (7315.5-11785.3)	0	345.4 (263.9-444.1)	0	0	0	0	0	0	0	0	0	0	0	0	0	0	0
Alcohol use	984.9 (466.3-1630.1)	0	9.7 (-5.9-27.2)	1764.4 (1247.9-2381)	0	1615.1 (1147.7-2221.6)	67.3 (37.8-106.7)	0	0	0	0	367.3 (249.1-508.0)	0	0	1954.4 (1033.8-2509.3)	233.6 (129.3-355.2)	482.1 (296.4-711.9)	254.1 (112.1-458.0)
Childhood sexual abuse and bullying	0	0	0	0	49.6 (18.2-101.8)	0	0	0	0	0	0	0	0	0	12.6 (1.4-32.7)	0	0	0
Low physical activity	132.8 (53.6-289.2)	0	9.1 (4.0-18.1)	0	0	5.7 (2.7-10.5)	0	0	0	0	0	0	0	0	0	0	0	0
Intimate partner violence	0	0	0	0	52.2 (0.2-123.3)	0	0	0	0.4 (0.03-1.4)	0	0	0	0	0	0	0	45.7 (26.6-74.1)	0
Low bone mineral density	0	0	0	0	0	0	0	0	0	0	0	0	0	0	0	45.2 (34.5-58.0)	3.6 (2.7-4.9)	96.2 (72.4-125.8)
Other environmental risks	185.7 (51.6-357.8)	0	5.7 (1.4-11.8)	0	13.3 (4.6-25.5)	26.7 (1.9-82.6)	0	0	0	0	0	0	0	0	0	0	0	0
Drug use	0	0	0	58.5 (28.1-112.6)	0	137.3 (68.8-252.6)	0	0	2.9 (0.3-9.3)	0	0	0	0	0	76.9 (54.1-102.7)	0	2.4 (1.1-4.3)	0
Occupational risks	0	42.2 (31.2-57.2)	0	0	0	20.1 (12.7-30.2)	0	0	0	0	0	0	201.2 (133.1-279.5)	64.1 (43.2-91.4)	0	10.8 (7.9-14.2)	0	15.5 (10.5-22.5)
Unsafe water, sanitationand handwashing	0	0	0	0	0	0	0	269.7 (168.8-434.8)	0	0	0	75.7 (31.3-131.3)	0	0	0	0	0	0
Unsafe sex	0	0	0	0	0	363.5 (262.7-510.0)	0	0	26.9 (14.9-43.5)	0	0	0	0	0	0	0	0	0
Non-optimal temperature	771.2 (386.2-1280.1)	51.3 (28.5-80.5)	40.3 (-14.7-117.6)	0	0	0	0	0	0	0	0	162.7 (33.1-323.3)	0	0	0			
High fasting plasma glucose	598.6 (393.1-888.6)	0	435.9 (338.5-550.1)	0	0	40.9 (9.8-94.7)	15.5 (2.0-54.6)	0	0	0	0	10.6 (6.1-16.2)	0	2.4 (0.5-5.9)	0	0	0	0
Kidney dysfunction	748.2 (528.4-1000.2)	0	471.9 (378.5-593.1)	0	0	0	0	0	0	0	0	0	1.0 (0.6-1.5)	0	0	0	0	0
High LDL cholesterol	1797.2 (1276.5-2402.4)	0	0	0	0	0	0	0	0	0	0	0	0	0	0	0	0	0
Alcohol use	15.3 (-96.5-141.4)	0		558.6 (388.7-764.4)	0	441.8 (277.0-656.4)	17.3 (7.6-33.1)	0	0	0	0	44.4 (21.9-72.6)	0	0	324.2 (250.6-412.8)	16.7 (6.5-30.4)	25.4 (8.8-47.7)	21.5 (8.0-41.5)
Tobacco	1004.2 (771.9-1296.8)	89.5 (64.5-120.8)	43.9 (21.8-68.2)	13.0 (8.5-19.2)	0	269.3 (187.4-375.7)	17.1 (5.4-42.2)	0	0	0	0	152.2 (92.3-222.8)	50.8 (32.5-73.0)	1.6 (1.0-2.4)	0	1.5 (1.0-2.2)	0.2 (0.1-0.3)	4.4 (2.7-6.7)
Child and maternal malnutrition	0	0	0	0	0	0	0	102.7 (37.4-241.9)	0	775.7 (609.8-973.8)	365.8 (233.4-546.4)	427.8 (275.7-609.3)	0	0	0	0	0	0
Air pollution	2245.6 (1723.9-2890.0)	109.6 (76.4-152.2)	58.7 (38.3-82.5)	0	0	72.1 (48.9-103.9)	0	1.8 (0.4-5.4)	0	110.7 (10.4-218.1)	0	225.7 (132.7-349.5)	0	19.3 (8.8-32.5)	0	0	0	0
High body-mass index	2395.3 (1456.3-3472.4)	38.7 (21.3-63.8)	317.2 (205.6-443.4)	49.8 (28.2-78.7)	0	423.9 (155.2-792.6)	51.5 (9.4-147.7)	0	0	0	0	0	93.6 (46.8-159.2)	4.0 (1.5-7.8)	0	0	0	0
Dietary risks	4119.2 (3228.3-5199.6)	0	137.9 (98.2-187.8)	0	0	266.6 (171.4-428.2)	0	0	0	0	0	0	0	0	0	0	0	0
High systolic blood pressure	4878.2 (3775.2-6220.8)	0	208.3 (152.5-276.6)	0	0	0	0	0	0	0	0	0	0	0	0	0	0	0

Numbers in brackets indicates 95% UI.

Annex 6. YLLs, YLDs and DALYs of GBD Level 3 causes attributable to the top 3 risk factors (A-Alcohol use, B- Dietary risk, C - High systolic blood pressure) in 2019

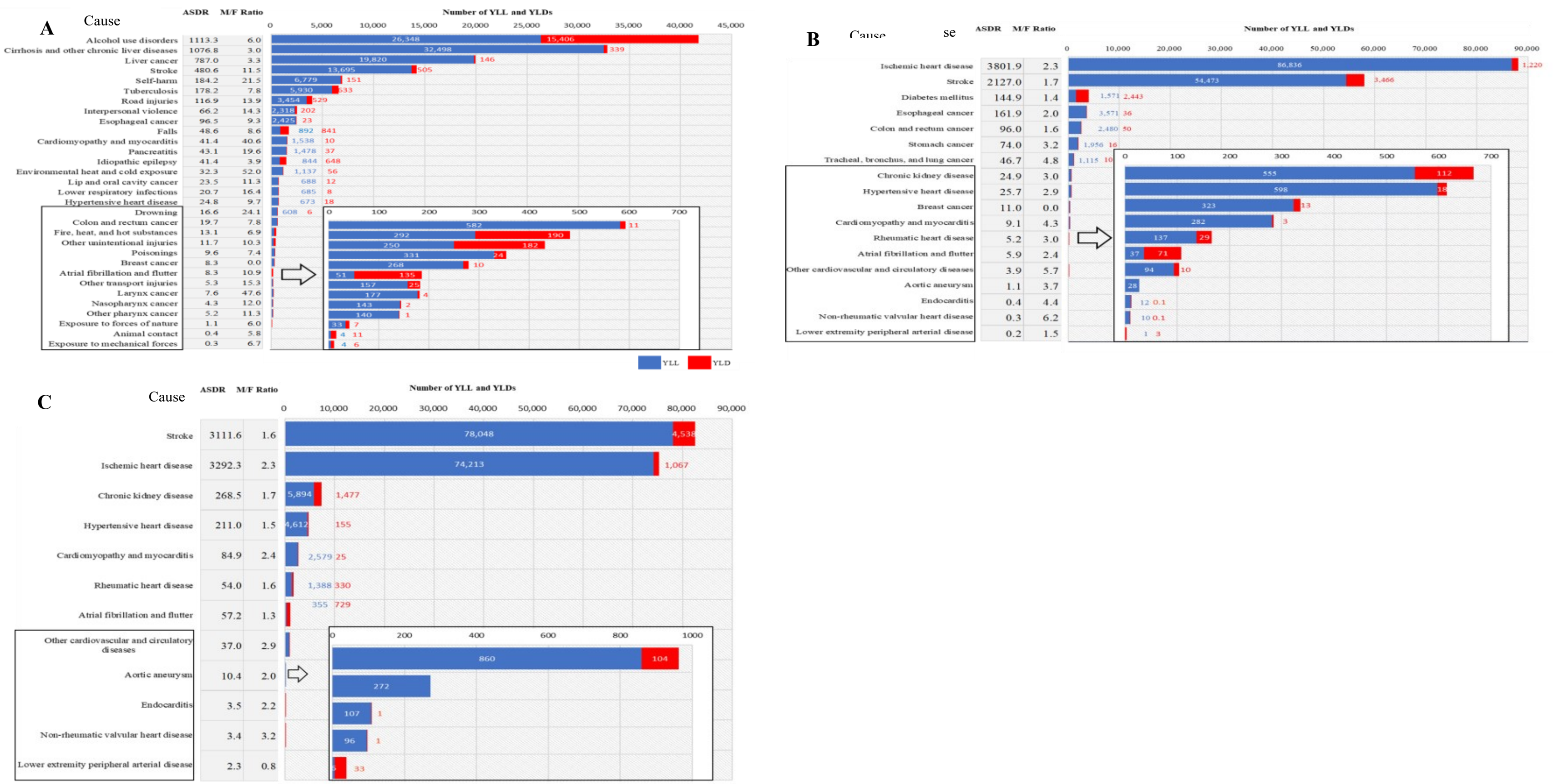

ASDR - Age-standardized rate of DALY

M/F Ratio - Male-to-female ratio

Annex 7. The burden of three top risk factors in Mongolia - All cause death, YLL, YLD and DALY, 2019

Risk factors[1] (Level 3)[2]	Measures	All ages			15-49				50-69				70+			
		#	AStR	M/F ratio	# (%)[3]		ASpR	M/F ratio	# (%)[3]		ASpR	M/F ratio	# (%)[3]		ASpR	M/F ratio
Alcohol use	Death	3,340	123.2	**4.6**	1,354	**(40.5)**	74.9	**7.4**	1,489	(44.6)	307.6	**4.6**	495	(14.8)	591.7	**3.4**
	YLL	121,914	3816.4	**5.4**	67,803	**(55.6)**	3750.9	**7.5**	46,485	(38.1)	9605.6	**4.8**	7,435	(6.1)	8890.6	**3.5**
	YLD	20,071	560.9	**5**	15,658	**(78.0)**	866.2	**4.4**	3,958	(19.7)	817.9	**7.3**	414	(2.1)	494.7	**7.9**
	DALYs	141,985	4377.3	**5.3**	83,461	**(58.8)**	4617.1	**6.7**	50,443	(35.5)	10423.6	**4.9**	7,849	(5.5)	9385.2	**3.6**
Dietary risk	Death	5,788	322.8	1.8	979	(16.9)	54.2	2.4	2,569	(44.4)	530.8	2.3	2,240	(38.7)	2678.9	1.5
	YLL	154,076	6265.2	2.1	45,874	(29.8)	2537.8	2.4	78,195	(50.8)	16158.2	2.4	30,007	(19.5)	35881.9	1.6
	YLD	7,499	275.0	1.1	2,627	(35.0)	145.3	0.9	3,796	(50.6)	784.5	1.2	1,075	(14.3)	1286.1	1.1
	DALYs	161,575	6540.2	2.0	48,502	(30.0)	2683.1	2.3	81,991	(50.7)	16942.7	2.3	31,083	(19.2)	37167.9	1.6
High blood pressure	Death	6,318	343.9	1.7	989	(15.7)	54.7	2.4	2,963	(46.9)	612.3	2.2	2,366	(37.4)	2828.8	1.4
	YLL	168,430	6819.8	2.0	46,093	(27.4)	2549.8	2.4	90,115	(53.5)	18621.3	2.2	32,223	(19.1)	38531.2	1.5
	YLD	8,459	316.3	1.2	2,801	(33.1)	154.9	1.1	4,352	(51.4)	899.3	1.3	1,306	(15.4)	1562.2	1.1
	DALYs	176,889	7136.2	1.9	48,894	(27.6)	2704.8	2.3	94,467	(53.4)	19520.6	2.2	33,529	(19.0)	40093.3	1.5

1) Top 2 risks (dietary risk and high systolic blood pressure) in terms of contribution to age-standardized mortality rate and 1 risk (alcohol use) with largest percentage change during 1990-2019
2) List of GBD Level 2 risk can be found in Reference# 10.
3) Percentage of total death, YLL, YLD and DALY due to respective risk factors
AStR - Age-standardized rate
ASpR - Age-specific rate
M/F - Male-to-Female
Numbers in Bold - Points emphasized in manuscript

Annex 8. 20 leading causes of YLDs with percentage changes from 1990 and 2019

MEN

Causes of Deaths[1]	YLD Rank 1990	YLD Rank 2019	YLD #	YLD % of all cause YLD	Age-standardized YLD rate per 100 000 (95% UI)	Age-standardized YLD rate % change 1990-2019 (95% UI)
Alcohol use disorders	6	1	12493.1	8.5	703.0 (478 to 999)	**98.0** (72.4 to 124.7)
Low back pain	2	2	11308.9	7.7	762.5 (538 to 1026)	-4.5 (-10.0 to 1.1)
Depressive disorders	3	3	8926.1	6.1	553.2 (379 to 763)	3.4 (-4.6 to 11.9)
Falls	7	4	7208.3	4.9	464.9 (325 to 657)	**26.7** (21.9 to 31.6)
Headache disorders	4	5	6979.2	4.7	414.7 (104 to 856)	-0.2 (-5.5 to 4.5)
Dietary iron deficiency	1	6	5850.9	4.0	355.4 (225 to 527)	**-39.3** (-52.2 to -23.0)
Age-related and other hearing lo	5	7	5712.7	3.9	473.5 (317 to 676)	**-4.8** (-9.3 to -0.6)
Exposure to mechanical forces	8	8	5014.2	3.4	316.9 (213 to 458)	**-5.6** (-10.1 to -1.2)
Oral disorders	9	9	4486.6	3.1	340.9 (205 to 531)	**-4.5** (-8.2 to -0.7)
Neonatal disorders	16	10	4088.0	2.8	227.8 (172 to 292)	**106.0** (49.3 to 172.9)
Road injuries	13	11	3862.9	2.6	246.9 (176 to 331)	**15.2** (11.2 to 19.4)
Schizophrenia	17	12	3224.5	2.2	182.5 (128 to 243)	4.4 (-7.6 to 17.9)
Blindness and vision impairmen	11	13	3204.1	2.2	312.0 (215 to 430)	**-16.8** (-21.4 to -12.1)
Stroke	18	14	3054.0	2.1	232.9 (166 to 296)	-5.9 (-13.0 to 1.7)
Dermatitis	10	15	2889.4	2.0	167.4 (94 to 269)	0.2 (-5.0 to 6.0)
Diabetes mellitus	32	16	2847.2	1.9	221.1 (143 to 311)	**63.1** (50.5 to 77.0)
Diarrheal diseases	12	17	2663.1	1.8	154.9 (106 to 219)	6.0 (-5.1 to 18.5)
Anxiety disorders	15	18	2541.8	1.7	155.4 (103 to 218)	1.4 (-9.4 to 13.3)
Drug use disorders	22	19	2501.2	1.7	140.8 (97 to 191)	**27.9** (10.3 to 49.5)
Idiopathic epilepsy	14	20	2437.6	1.7	151.7 (38 to 302)	9.8 (-72.1 to 382.7)
Sub total	NA		101293.9	68.9	NA	
All cause Total			146984.4	100.0	9707.7 (7221 to 12551)	-1.3 (-2.0 to 4.0)

WOMEN

Causes of Deaths[1]	YLD Rank 1990	YLD Rank 2019	YLD #	YLD % of all cause YLD	Age-standardized YLD rate per 100 000 (95% UI)	Age-standardized YLD rate % change 1990-2019 (95% UI)
Low back pain	2	1	14292.0	8.3	873.0 (617 to 1165)	-4.9 (-9.9 to 0.5)
Depressive disorders	1	2	13946.2	8.1	826.9 (563 to 1148)	-3.7 (-10.7 to 4.2)
Gynecological diseases	3	3	13652.1	8.0	727.2 (494 to 1022)	**-4.6** (-8.3 to -0.9)
Headache disorders	4	4	12604.7	7.4	714.0 (160 to 1511)	0.3 (-3.6 to 4.2)
Age-related and other hearing los	7	5	6273.1	3.7	451.1 (305 to 635)	**-4.1** (-8.5 to -0.2)
Oral disorders	8	6	5965.7	3.5	393.0 (246 to 588)	**-3.8** (-7.2 to -0.7)
Dietary iron deficiency	5	7	5910.3	3.4	345.9 (218 to 522)	**-44.3** (-57.1 to -30.7)
Dermatitis	6	8	5551.0	3.2	321.0 (182 to 532)	0.5 (-3.1 to 4.1)
Endocrine, metabolic, blood, and immune disorders	10	9	4783.8	2.8	274.7 (181 to 391)	-2.9 (-10.2 to 4.4)
Falls	14	10	4474.2	2.6	273.1 (191 to 385)	**14.8** (9.8 to 20.0)
Blindness and vision impairment	11	11	4281.9	2.5	311.5 (212 to 440)	**-14.4** (-18.4 to -9.8)
Anxiety disorders	9	12	3945.7	2.3	252.3 (167 to 358)	2.2 (-8.1 to 13.2)
Stroke	12	13	3942.7	2.3	260.7 (183 to 331)	**-22.5** (-28.5 to -16.2)
Neonatal disorders	17	14	3717.6	2.2	208.3 (154 to 269)	**94.3** (41.4 to 162.4)
Other muscoloskeletal disease	19	15	3627.4	2.1	215.1 (139 to 316)	**34.0** (16.7 to 57.8)
Diabetes mellitus	30	16	3126.4	1.8	208.4 (137 to 295)	**50.3** (40.1 to 61.6)
Schizophrenia	18	17	3123.9	1.8	168.1 (115 to 222)	3.8 (-7.8 to 16.7)
Osteoarthritis	21	18	2917.6	1.7	210.3 (106 to 421)	3.8 (0.0 to 7.9)
Alcohol use disorders	24	19	2913.3	1.7	155.5 (104 to 226)	**48.5** (26.6 to 74.9)
Diarrheal diseases	13	20	2804.1	1.6	161.3 (108 to 227)	5.0 (-4.7 to 17.2)
Sub total	NA		#######	71.1	NA	
All cause Total			#######	100.0	10404 (7691 to 13517)	**-5.0** (-7.0 to -2.0)

YLD= years lived with disability; UI= uncertainty interval; Changes in bold are statistically significant.

Annex 9. Age-standardized rate of death and YLLs attributable to different risk factors in Mongolia 2019

#	Risk factors	Deaths			YLLs		
		Men	Women	% Difference	Men	Women	% Difference
1	Air pollution	210.8	118.0	78.7	5064.0	2647.1	91.3
2	Alcohol use	220.2	47.6	**362.5**	6769.1	1258.8	**437.7**
3	Child and maternal malnutrition	18.8	14.1	33.6	1663.4	1230.9	35.1
4	Dietary risks	440.6	243.4	81.0	8858.4	4262.9	107.8
5	Drug use	27.1	8.7	**209.4**	757.9	205.7	**268.4**
6	High body-mass index	195.4	125.1	56.2	5103.4	2924.6	74.5
7	High fasting plasma glucose	86.3	46.3	86.2	1718.5	841.8	104.1
8	High LDL cholesterol	192.6	103.5	86.1	4053.2	1726.6	134.8
9	High systolic blood pressure	452.0	267.3	69.1	9392.0	4794.1	95.9
10	Kidney dysfunction	93.2	54.5	70.8	1992.7	1077.8	84.9
11	Low bone mineral density	4.8	2.0	143.8	138.0	49.2	180.4
12	Low physical activity	20.8	12.1	71.9	272.2	136.6	99.3
13	Non-optimal temperature	69.4	48.6	42.9	954.6	849.0	12.4
14	Occupational risks	14.8	2.7	**446.7**	439.7	64.3	**583.9**
15	Other environmental risks	38.1	13.4	**184.0**	671.9	208.5	**222.2**
16	Unsafe sex	0.3	12.4	-97.8	9.8	363.6	-97.3
17	Unsafe water, sanitation, and handwashing	2.8	2.8	0.0	159.1	199.0	-20.1

Risk factors analyzed are GBD Level 2 risk factors

Numbers in Bold - Points emphasized in manuscript
